# Complementary omics strategies to dissect p53 signaling networks under nutrient stress

**DOI:** 10.1007/s00018-022-04345-8

**Published:** 2022-05-30

**Authors:** Markus Galhuber, Helene Michenthaler, Christoph Heininger, Isabel Reinisch, Christoph Nössing, Jelena Krstic, Nadja Kupper, Elisabeth Moyschewitz, Martina Auer, Ellen Heitzer, Peter Ulz, Ruth Birner-Gruenberger, Laura Liesinger, Georgia Ngawai Lenihan-Geels, Moritz Oster, Emil Spreitzer, Riccardo Zenezini Chiozzi, Tim J. Schulz, Michael Schupp, Tobias Madl, Albert J. R. Heck, Andreas Prokesch

**Affiliations:** 1 Gottfried Schatz Research Center for Cell Signaling, Metabolism and Aging, Division of Cell Biology, Histology and Embryology, Medical University of Graz, 8010 Graz, Austria; 2 Cancer Research UK Beatson Institute, Garscube Estate, Glasgow, UK; 3 Diagnostic and Research Institute of Human Genetics, Medical University of Graz, 8010 Graz, Austria; 4 Diagnostic and Research Institute of Pathology, Medical University of Graz, 8010 Graz, Austria; 5 Institute of Chemical Technologies and Analytics, Technische Universität Wien, 1060 Vienna, Austria; 6 Department of Adipocyte Development and Nutrition, German Institute of Human Nutrition Potsdam-Rehbrücke, Nuthetal, Germany; 7 Institute of Pharmacology, Charité-Universitätsmedizin Berlin, Corporate member of Freie Universität Berlin and Humboldt Universität Zu Berlin, 10115 Berlin, Germany; 8 Gottfried Schatz Research Center for Cell Signaling, Metabolism and Aging, Division of Molecular Biology and Biochemistry, Medical University of Graz, 8010 Graz, Austria; 9 Biomolecular Mass Spectrometry and Proteomics, Bijvoet Center for Biomolecular Research, Utrecht Institute of Pharmaceutical Sciences, Utrecht University, 3584CH Utrecht, The Netherlands; 10 Netherlands Proteomics Center, 3584CH Utrecht, The Netherlands; 11 German Center for Diabetes Research (DZD), Munich-Neuherberg, Germany; 12 Institute of Nutritional Science, University of Potsdam, Potsdam-Rehbrücke, Nuthetal, Germany; 13 BioTechMed-Graz, 8010 Graz, Austria

**Keywords:** p53 signaling, Nutrient stress, Starvation, Interactome, p53 targets

## Abstract

Signaling trough p53is a major cellular stress response mechanism and increases upon nutrient stresses such as starvation. Here, we show in a human hepatoma cell line that starvation leads to robust nuclear p53 stabilization. Using BioID, we determine the cytoplasmic p53 interaction network within the immediate-early starvation response and show that p53 is dissociated from several metabolic enzymes and the kinase PAK2 for which direct binding with the p53 DNA-binding domain was confirmed with NMR studies. Furthermore, proteomics after p53 immunoprecipitation (RIME) uncovered the nuclear interactome under prolonged starvation, where we confirmed the novel p53 interactors SORBS1 (insulin receptor signaling) and UGP2 (glycogen synthesis). Finally, transcriptomics after p53 re-expression revealed a distinct starvation-specific transcriptome response and suggested previously unknown nutrient-dependent p53 target genes. Together, our complementary approaches delineate several nodes of the p53 signaling cascade upon starvation, shedding new light on the mechanisms of p53 as nutrient stress sensor. Given the central role of p53 in cancer biology and the beneficial effects of fasting in cancer treatment, the identified interaction partners and networks could pinpoint novel pharmacologic targets to fine-tune p53 activity.

**Supplementary Information:**

The online version contains supplementary material available at 10.1007/s00018-022-04345-8.

## Introduction

The tumor suppressor p53 is a multifunctional protein involved in various cellular stress responses [1]. Acting either as transcription factor or through protein–protein interactions [2], p53 can coordinate downstream cell fate decisions to maintain genomic stability, control cell cycle progression, induce cell death or senescence, modulate autophagy, and determine cell differentiation and pluripotency [3, 4]. Coinciding with inclusion of inflammation and metabolism as central hallmarks of cancer [5], recent reports implicate p53 in the regulation of immune responses [6] and of various metabolic pathways involved in the management of all major classes of macromolecules (i.e. glucose, amino acids, and lipids; [7, 8]). To coordinate these fine-tuned activities, cellular p53 levels need to be regulated in defined margins, as loss-of-function is often detrimental (tumorigenic) and hyperactivation can lead to excessive cell death [9, 10].

A key determinant of p53 activation in cells is the interplay between p53 and MDM2. In fact, MDM2 can act as both inhibitor and activator of p53. Functioning as a p53 inhibitor, MDM2 directly binds to the N-terminal region of p53, causing p53 ubiquitination followed by p53 nuclear export, proteasomal degradation, or transactivation potential inhibition [11, 12]. Certain post-translational modifications of MDM2, however, stimulate binding to and stabilization of p53 mRNA, which cumulates into p53 activation [13, 14]. Adding to the regulatory complexity, MDM2 is a major transcriptional target of p53, forming a negative feedback loop to constrain cellular p53 protein abundance under conditions favoring MDM2-mediated p53 inhibition [15, 16]. Of note, in addition to MDM2, p53 stability can be influenced through interaction with many other proteins [17] and numerous post-translational modifications [18].

Once activated, p53 can regulate cell fate through protein–protein interactions (e.g. regulating glucose flux by binding to G6PD in cancer cells [19]) or through acting as transcription factor to directly activate hundreds of target genes in various pathways [20, 21]. Determinants of p53 selectivity and affinity on genomic binding sites include cellular p53 levels and expression dynamics, p53 post-translational modifications, chromatin state, and interaction with nuclear cofactors (elegantly reviewed in [22]). The context-dependent selection of a specific ensemble of target genes by p53 as well as the upstream cues and specific stresses leading to p53 activation are ongoing subjects of intense research.

While fasting regimens have shown health benefits in many pathologies [23, 24] and are currently undergoing clinical testing as cancer adjuvant therapy [25–27]), from the viewpoint of a single cell, starvation constitutes a severe stress. Consistent with its role as stress response factor, we recently reported that p53 is stabilized in starved HepG2 cells and in mouse and human primary hepatocytes [28], and that p53 is necessary and sufficient for the therapy-enhancing effect of fasting on sorafenib treatment in hepatocellular carcinoma models [29]. Moreover, hepatic p53 was recently shown to be stabilized by O-GlcNAcylation in fasted mice, which was shown to be essential for the regulation of gluconeogenesis in the liver [30]. Here we investigate the upstream regulatory events that activate p53 signaling upon starvation, as well as the chromatin-level interactions and transcriptional programs that define the downstream activities of p53 as a starvation response hub. We show that immediate-early dissociation of MDM2 and p53 leads to sustained stabilization of nuclear p53. A proximity-based labeling proteomics approach (BioID) identified p53 interactors early upon nutrient withdrawal and suggests the kinase PAK2 as directly interacting p53 regulator. Downstream, nuclear p53 is associated with a network of chromatin modifiers and other nuclear factors to mount a defined transcriptional starvation response, regulating canonical as well as yet unknown p53 target genes. Hence, using complementary mass spectrometry (MS)- and sequencing-based omics technologies we reveal p53 as a central node in the cellular response to nutrient withdrawal.

## Results

### Starvation dissociates p53-MDM2 regulatory feedback loop leading to robust nuclear p53 stabilization

**Fig. 1:**
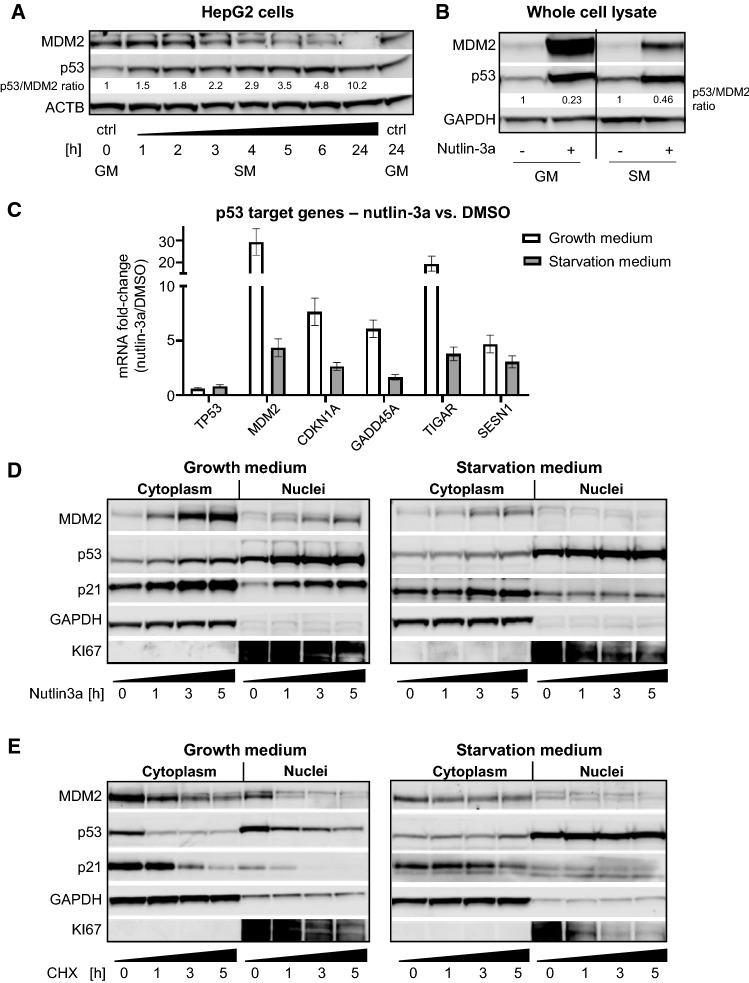
Starvation dissociates the p53-MDM2 regulatory feedback loop leading to robust nuclear p53 stabilization. **A** Western blot from a time course experiment in HepG2 cells showing p53 and MDM2 protein abundances over 24 h starvation. Densiometric quantification for ratio of p53/MDM2 band intensity is indicated with black numbers below bands. Beta-actin (ACTB) as loading control. GM growth medium, SM starvation medium. **B** Western blot showing regulation of p53 and MDM2 after 24 h GM or SM with or without pharmacological inhibitor nutlin-3a (10 µM). Densiometric quantification for ratio of p53/MDM2 band intensity is indicated with black numbers below bands. GAPDH as loading control. **C** RT-qPCR showing fold-change of p53, MDM2, CDKN1A (p21), GADD45a, TIGAR, and SESN1 mRNA expression levels in 6 h nutlin-3a treated samples over vehicle (DMSO) controls after preincubation for 24 h with growth medium or starvation medium. Shown are replicate measurements of one representative out of three independent experiments. Data are shown as mean ± SEM. **D** Western blot of p53, MDM2, and p21. HepG2 cells were kept in growth medium (left panel) or starvation medium (right panel) for 24 h before they were treated with nutlin-3a (10 µM) for the indicated times. Loading controls: cytoplasm GAPDH, nuclei KI67. **E** Western blot of protein degradation assay showing regulation of p53, MDM2, and p21. HepG2 cells were kept in growth medium (left panel) or starvation medium (right panel) for 24 h before they were treated with cycloheximide (CHX) for the indicated times. Loading controls: cytoplasm GAPDH, nuclei KI67.

p53 is key in integrating extracellular cues, such as nutrient stress, to regulate cell fate either through transcriptional mechanisms or through protein–protein interactions [3, 4]. A central determinant of p53-mediated cellular outcome is its interaction with its main endogenous inhibitor MDM2 [31]. Extending our previously published results that indicated stabilized p53 protein in response to starvation in HepG2 cells [28], we conducted a time series experiment to probe p53 and MDM2 protein dynamics after exposure to starvation medium [28, 32]. While p53 protein was immediately (after 1 h of starvation medium) and increasingly induced, MDM2 protein abundance declined reciprocally to very low levels at 24 h of starvation (Fig. 1A and Fig S1A), whereas mRNA levels of both p53 and MDM2 did not reflect protein abundances (Fig S1B). As both a transcriptional target and major negative regulator of p53, MDM2 is described to elicit a negative feedback loop to constrain p53 induction, as very high p53 levels lead to apoptosis in many cell systems [33]. In our system, however, sustained p53 levels after incubation in starvation medium for 24 h did not lead to reduced cell viability (Fig S1C). The continuous reduction of MDM2 suggests disruption of the p53/MDM2-feedback loop by starvation as an underlying mechanism of sustained p53 stabilization. To test this, we reasoned that the effect of nutlin-3a should be dampened in starvation conditions. The small molecule nutlin-3a is a highly specific and potent inhibitor of the MDM2/p53-interaction, leading to p53 protein stabilization and target gene activation [34]. Accordingly, nutlin-3a under starvation conditions led to diminished induction of MDM2 protein (Fig. 1B and S1D) and to blunted mRNA fold-changes of known p53 target genes (Fig. 1C), when compared to nutlin-3a responses in full growth medium. In line with this, p53 co-precipitates with MDM2 exclusively in cells grown in growth medium and not in starvation medium (Fig S1E).

p53 transactivation potential is regulated largely through shuttling between the cytoplasm and nucleus [35]. To investigate p53/MDM2 dynamics in cellular compartments we performed fractionation experiments following three time points of nutlin-3a treatment after keeping cells either in growth medium or starvation medium for 24 h. While in growth medium nuclear p53 protein as well as p53 targets MDM2 and p21 increase with time, these nutlin-3a responses were severely blunted in starvation medium (Fig. 1D), again indicating disruption of the p53/MDM2 feedback loop under starvation.

To further investigate prolonged nuclear stabilization of p53 in starvation medium we performed a protein degradation assay with the translation inhibitor cycloheximide (CHX) after keeping the cells in growth or starvation medium for 24 h. As shown in Fig. 1E, in growth medium p53 is subject to rapid degradation following 1 h of CHX treatment, commensurate with earlier reports [36]. After 24 h in starvation medium, when p53 is mainly localized in the nucleus, it is markedly stabilized as no degradation could be detected after 5 h of CHX. This is likely owed to the nuclear exclusion (Fig. 1E) and proteasomal degradation (Fig S1F) of MDM2 in starvation conditions. Nuclear localization upon starvation versus growth medium was also confirmed by immunofluorescence imaging (Fig S1G).

Together, these data indicate that starvation conditions disrupt the p53/MDM2 regulatory cycle, which leads to robust and sustained nuclear stabilization of the p53 protein.

### Proximity-based labeling proteomics reveals the p53 interactome under starvation

**Fig. 2:**
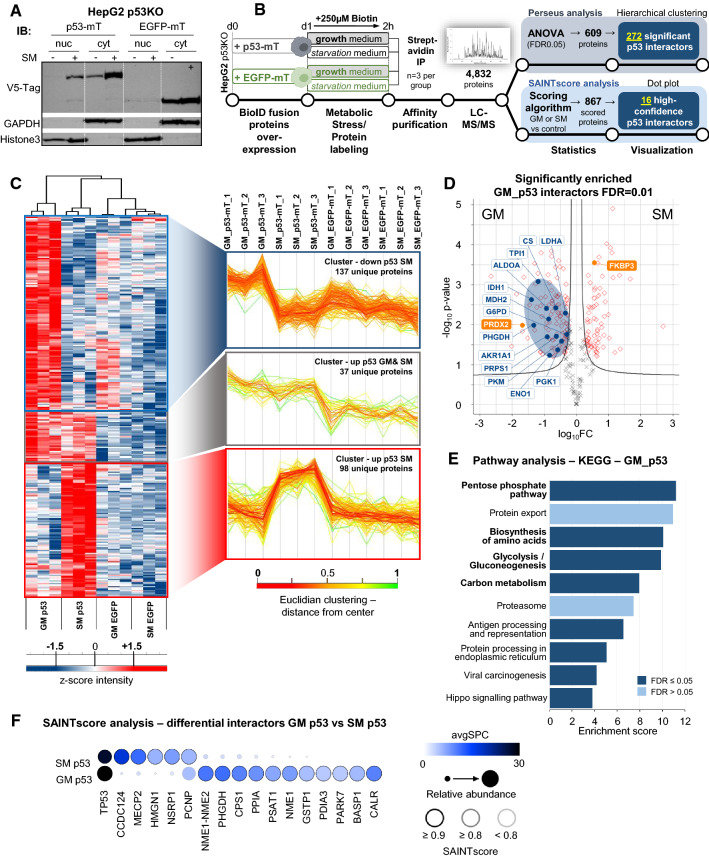
Proximity-based biotin labeling proteomics reveals specific p53 interactome changes upon starvation. **A** Western blot showing HepG2 p53KO cells overexpressing either p53-miniTurbo or EGFP-miniTurbo fusion protein. Cells were subjected to growth medium (GM) or starvation medium (SM) and fractionated into nuclei (nuc) and cytoplasm (cyt). Overexpressed proteins were detected with a V5-Tag-specific antibody. GAPDH (cytoplasm), Histone3 (nuclei) as loading controls. **B** Proximity-biotinylation and affinity purification MS experimental and analysis workflow. **C** Left: Hierarchical clustering of proteomics data after ANOVA testing (FDR0.05)/z-scoring, showing three clusters with p53-dependently enriched signals over EGFP background control. Right: Profile blots showing protein abundances within clusters between biological replicates (*n* = 3 per group). Top profile: enriched p53 interactors under GM conditions (137 proteins). Middle profile: enriched p53 interactors under both, GM and SM, conditions (37 proteins). Bottom profile: enriched p53 interactors under SM conditions (98 proteins). Total 272 proteins. **D** Volcano bot showing 272 proteins enriched over EGFP background control. Proteins with significant fold-changes between GM and SM conditions are located outside the cut-off curve (red diamonds) (FDR0.01|S0 = 0.1; Perseus). Significantly changed proteins were subjected to KEGG pathway overrepresentation analysis (**E**) and proteins from overrepresented pathways (pentose phosphate pathway, biosynthesis of amino acids, glycolysis/gluconeogenesis, carbon metabolism) labelled with protein names and circled in blue. Proteins that reportedly influence p53 stability by modifying ubiquitination are marked in orange. **E** KEGG pathway overrepresentation analysis (WebGestalt) with proteins significantly changed between GM and SM. **F** SAINT score analysis of differential p53 interactors GM vs SM. Cut-offs SAINT probability: (SP) ≥ 0.9; SP ≥ 0.8; SP < 0.8 indicated with grey scaled circles. Relative abundance is represented by circle diameter. Blue tones indicate the respective average spectral counts (avgSPC).

To gain further insight into the p53-regulating mechanisms in starved cells we established a BioID-based proteomics approach to determine the cytoplasmic p53 interactome early upon nutrient withdrawal. Therefore, we cloned the p53 coding sequence in an expression vector coding for an N-terminal V5-tag and a biotin ligase with a nuclear export sequence (NES) fused to the p53 C-terminus. The miniTurbo (mT) biotin ligase is optimized for minimal steric hindrance and short labeling time [37]. As a background labeling control, we cloned the coding sequence for EGFP instead of p53 into the fusion vector. To perform the overexpression on a background with non-detectable endogenous p53, we used CRISPR/Cas9 to knock out p53 (p53KO) in HepG2 cells (Fig S2A). Pilot experiments established efficient biotin labeling of cellular proteins with 250 µM exogenous biotin supplemented to the medium (Fig S2B) within a 2 h period, before p53 is enriched in nuclei (Fig S2C). Immunofluorescence microscopy after transfection in these p53KO cells validated cytoplasmic location of both fusion proteins (Fig S2D). Importantly, when performing subcellular fractionation followed by Western blot, we observed a clear enrichment of cytoplasmic p53 protein under starvation (Fig. 2A). Hence, we overexpressed either p53-mT or EGFP-mT fusion protein in HepG2 p53KO cells and subjected the cells to either growth medium or starvation medium for 2 h, both supplemented with biotin (Fig. 2B). As proteins in close proximity to p53-mT are biotin labelled in a cumulative manner, we reasoned that the time frame of 2 h should facilitate identification of immediate-early cytoplasmic interactions (Fig S2C) upon change to starvation medium. Subsequently, the cell lysate was enriched for biotin labeled proteins through streptavidin pull-down and the enriched material was analyzed with MS (Fig. 2B). After peptide mapping, hierarchical clustering identified three clusters with proteins specifically enriched in the p53 group over the EGFP control (Fig. 2C, green marked in Fig S2E). p53 is found in growth and starvation medium samples with a high coverage of 36 peptides (Table S1), as would be expected as a consequence of auto-biotinylation of the fusion protein (Fig S2B marked with asterisks). Performing statistical analysis on these three clusters defines 115 and 75 proteins as potential p53 interactors in growth medium and starvation medium, respectively (FDR = 0.01; Fig. 2D). The abundance of proteins in non-transfected negative controls does not significantly change between growth medium or starvation conditions (Fig S2F). Examining the list of significantly enriched proteins (Table S1), we noticed a number of known p53 interactors. Firstly, several members of the high-mobility protein group (HMG) family are detected specifically in starvation medium. In particular, HMGB1 known to directly bind to p53 [38] shows a high peptide abundance (27 peptides) in our MS data, indicating intricate interaction with p53 under starvation conditions. Secondly, FKBP prolyl isomerase 3 (FKBP3), found enriched upon starvation (Fig. 2D), was described to interact with MDM2 to promote its proteasomal degradation (as shown in Fig S1D under starvation) and p53 stabilization [39]. Additionally, Peroxiredoxin-2 (PRDX2), recently reported as promoting p53 ubiquitination and degradation [40], was found de-enriched upon starvation (Fig. 2D). Moreover, p53 and its negative regulators MDM2/MDMX have been shown to be regulated by the 14-3-3 protein interaction network [41] and we found several members of the adapter protein family 14-3-3 (gene names: YWHAB, YWHAE, YWHAG, YWHAQ, YWHAZ) with high sequence coverage under growth medium as compared to starvation medium conditions, suggesting dissociation of the 14-3-3/p53 complex upon nutrient withdrawal.

Performing KEGG pathway over-representation analysis on p53 interactors under growth medium conditions yielded enrichment of proteins of metabolic pathways such as the pentose phosphate pathway (PPP), glycolysis, amino acid biosynthesis, and carbon metabolism (Fig. 2E, proteins in significant metabolic pathways marked are in Fig. 2D as blue circles). In fact, mapping EC numbers to proteins in all three clusters shows a significant enrichment of enzymes in the growth medium group (Fisher’s exact test, *p* = 0.0101; Fig S2E). Our data set identified several p53-associated enzymes, including key metabolic enzymes such as Phosphoglycerate kinase 1 (PGK1), Citrate synthase (CS), Malate dehydrogenase (MDH2), l-lactate dehydrogenase A chain (LDHA), Glucose-6-phosphate 1-dehydrogenase (G6PD), Ribose-phosphate pyrophosphokinase 1 (PRPS1), and Pyruvate kinase (PKM). Intriguingly, we found two members of the very small group of metabolic enzymes that are altered in cancers in the list of potential p53 interactors in growth medium: D-3-phosphoglycerate dehydrogenase (PHGDH) [42, 43] and Isocitrate dehydrogenase [NADP] cytoplasmic (IDH1), the latter of which is involved in the regulation of the oncometabolite d-2-hydroxylglutarate [44]. Furthermore, our approach identified a number of mitochondrial proteins, such as components of the electron transport chain, as putative p53 interactors under starvation. Most noticeably, transcription factor A (TFAM) [45], the master regulator of mitochondrial DNA replication, is significantly enriched under starvation conditions with a high peptide coverage (47%). To identify more stringently controlled true positive p53 interactions, we utilized the Significance Analysis of Interactome (SAINT) algorithm (Fig. 2F) [46–48]. Overall, 16 high-confidence p53 interactors surpassed a stringent SAINT probability (SP) cut-off ≥ 0.9. Strikingly, we found PHGDH robustly scored as interactor in growth medium conditions, confirming our finding with hierarchical clustering. PHGDH (SP = 0.96), which controls glycolytic flux into serine and glycine synthesis has been described as a direct p53 target gene [43], but we are the first to identify direct protein–protein interaction. Furthermore, another enzyme mediating de novo serine synthesis, Phosphoserine aminotransferase (PSAT1) (SP = 0.93), is enriched in the growth medium group.

While these novel, putative p53 interaction partners need verification with complementary methods, they harbor the potential to extend our knowledge of the functional spectrum of p53 in the cellular response to nutrient changes and as a metabolic regulator [10, 49, 50] of serine biosynthesis and oncometabolite production.

### Differential peptide mapping reveals serine/threonine-protein kinase PAK2 as upstream regulator directly binding to p53

**Fig. 3:**
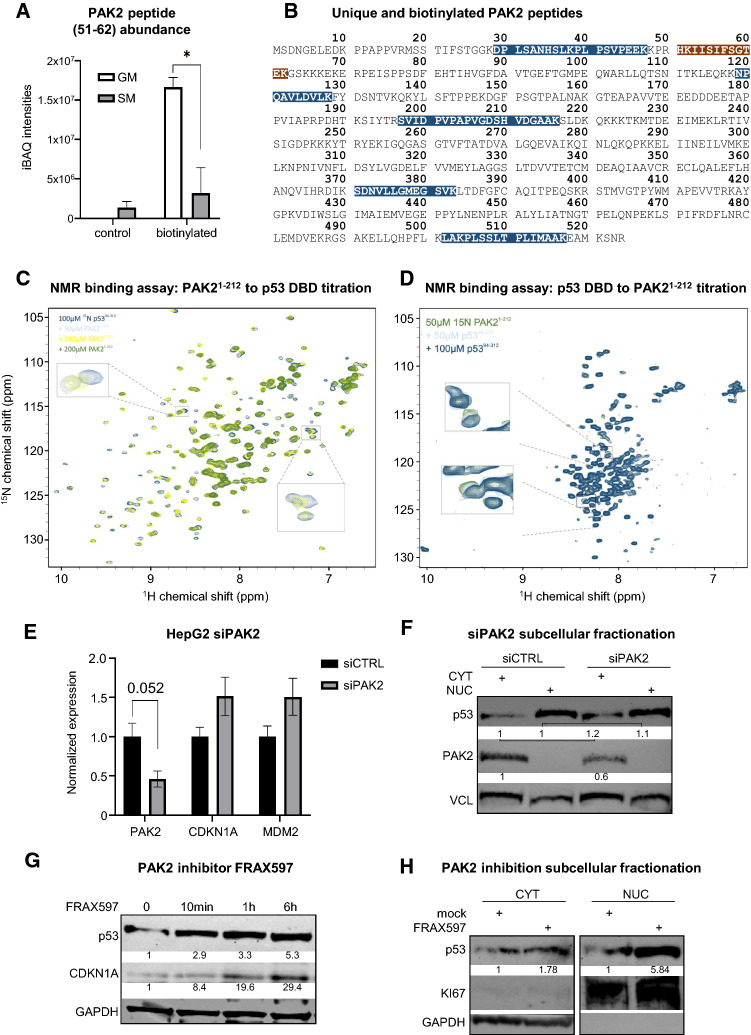
Differential peptide mapping reveals serine/threonine-protein kinase PAK2 as upstream regulator directly binding to p53. **A** Bar graph showing iBAQ intensities of PAK2 peptide 51–62 in untreated samples (control) vs biotin treated samples. Biotinylation on K52 was highly increased under growth medium (GM) conditions, while it significantly declined under starvation medium (SM) conditions. Data are shown as mean ± SEM. Student’s *t* test, *p < 0.05. **B** PAK2 protein sequence with indicated peptides uniquely identified in p53 samples (GM or SM) but absent in EGFP control samples (blue boxes). Highly significant differentially biotinylated peptide between GM and SM conditions (shown in **A**) (brown box). **C** ^1^H, ^15^N HSQR NMR spectra of ^15^N-labelled recombinant p53 DBD (94–312) incubated with increasing concentrations of recombinant PAK2 N-terminal domain (1–212). Insets show clear concentration-dependent chemical shift perturbations. **D** ^1^H, ^15^N HSQR NMR spectra of ^15^N-labelled N-terminal PAK2 domain (1–212) incubated with increasing concentrations of p53 DBD (94–312). Insets show clear concentration-dependent chemical shift perturbations. **E** RT-qPCR showing p53 target gene activation upon PAK2 silencing in HepG2 cells. Student’s *t* test, *p*-values are indicated. **F** Western blot showing PAK2 knockdown after transfection with siPAK2 and non-targeting control (siCTRL). Densiometric quantifications (black numbers) under knockdown conditions (siPAK2) are related to protein levels under control conditions. Vinculin (VCL) as loading control. **G** Western blot showing a time course experiment with PAK2 inhibitor FRAX597 (20 nM) over 6 h of treatment and concomitant CDKN1A (p21) accumulation. Densiometric quantifications are indicated with black numbers below bands. GAPDH as loading control. **H** Western blot showing PAK2 inhibition with FRAX597 (1 µM) and resulting p53 accumulation in cytoplasm and nuclei. Densiometric quantifications are indicated with black numbers below bands. GAPDH (cytoplasm) and KI67 (nuclei) loading controls

Through mapping several MS-detected peptides to protein amino acid sequences, information of single peptide occurrences and abundances, and therefore domain-specific interactions, might be obscured. Thus, we performed a complementary analysis on the level of unique peptides that are present exclusively in p53 overexpressing samples and identified 1050 peptides (Table S2). Additionally, a BioID approach allows for detection of differentially biotinylated peptides within the generated MS spectra. This analysis revealed 17 biotinylated peptides exclusively present in the p53 versus EGFP samples (Table S2). Focusing on proteins that can modify protein activity by posttranslational mechanisms, among the peptides differentially biotinylated under growth but not starvation medium we noticed one peptide mapping to the serine/protein kinase PAK2 (Fig. 3A, B; Table S2). Strikingly, also among the 1050 unique peptides found in the p53 groups, 9 peptides correspond to PAK2 or its activator, Cell division control protein 42 homolog (CDC42, [51]) (Table S2). Interestingly, overlapping the BioGRID [52] interactome of PAK2 with the list of growth medium-enriched p53 interactors (Fig. 2D) yields four 14-3-3 proteins and the Ras-related C3 botulinum toxin substrate 1 (RAC1), another well-described PAK2 activator, as common interactors (Fig S3A) [53]. Thus, we provide evidence for a close association of p53, PAK2 and its activators, and 14-3-3 proteins under growth medium conditions, which is corroborated by prediction of 14-3-3 protein binding sites in both PAK2 and p53 (Fig S3B) [54].

To address the question whether PAK2 and p53 indeed physically interact and to determine which domains mediate these interactions we employed nuclear magnetic resonance (NMR) spectroscopy. Since the differentially biotinylated PAK2 peptide is localized within the N-terminus (Fig. 3B, Table S2), we titrated a recombinant PAK2 N-terminal domain [amino acids (aa) 1-212] to a solution containing either ^15^*N*-labeled p53 DNA binding domain (DBD; aa 94-312) or p53 transactivation domain (TAD; aa 1-94). This titration revealed a progressive chemical shift perturbation (CSP) and progressive loss of signal intensity of ^1^H, ^15^N HSQC cross peaks with the p53 DBD (Fig. 3C and S3C), while no CSPs were observed upon testing ^15^*N*-labeled p53 TAD (Fig S3D). Inversely, titration of the p53 DBD to ^15^*N* labeled PAK2 *N*-terminal domain induced complementary CSPs (Fig. 3D). Together, these results indicate that the PAK2 N-terminal domain binds specifically to the p53 DBD in a concentration-dependent manner.

Since PAK2 is a potential upstream regulator of p53, we reasoned that PAK2 inhibition would represent a stimulus that, at least in part, mimics nutrient deprivation and translates into activation of p53 signaling. Indeed, knock down of PAK2 (Fig. 3E, F) in HepG2 cells led to a trend in induction of expression of canonical p53 target genes (Fig. 3E) and to a slight induction of p53 protein (Fig. 3F). Next, we used pharmacological PAK inhibition utilizing the group I PAK-specific kinase inhibitor FRAX597 [55] in HepG2 cells kept in growth medium. This led to p53 accumulation and gradual p21 upregulation over 6 h of treatment (Fig. 3G) and to increase of p53 protein levels, particularly in the nuclear fraction (Fig. 3H). Based on our findings and previous reports [56], we propose a model (Fig S3E) where PAK2/p53 interaction (maybe through PAK2-dependent p53 phosphorylation) renders p53 prone to degradation (p53 “off state”), while starvation-induced disruption of this complex (p53 “on state”) leads to p53 stabilization followed by nuclear translocation and p53 activation. However, we note that the changes in p53 activity upon PAK2 modulation are smaller than after a starvation stimulus (Fig. 1). This is most likely due to the pleiotropic effects of starvation and that PAK2/p53 dissociation is only one of several mechanism impinging on p53 signaling during starvation [22].

Nevertheless, our peptide-based analyses in conjunction with robust NMR results identified PAK2 as a potential p53 interactor and as an immediate-early regulator of cellular p53 levels upon nutrient withdrawal.

### Nuclear p53 interactome in starved cells

**Fig. 4:**
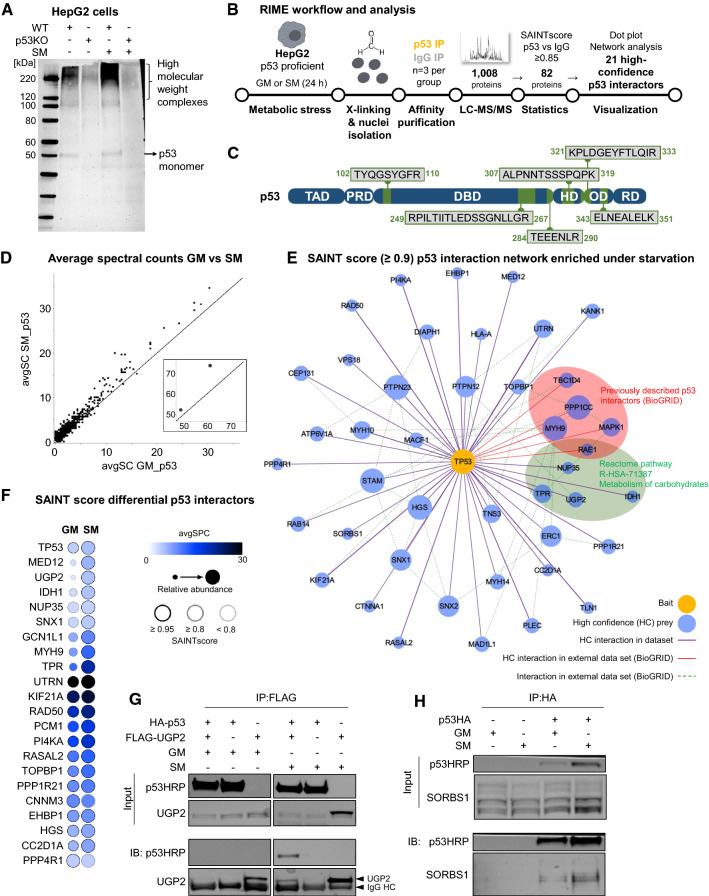
Nuclear p53 interactome under prolonged starvation: SORBS1 and UGP2 as novel interactors. **A** Western blot probed with p53 antibody (DO1) showing high molecular weight complexes (HMWC) specific for HepG2 wt cells, while p53KO cells lack p53-dependent staining in the high molecular weight area of the immunoblot. Starvation (SM) leads to a p53-dependent increase of HMWC in comparison to growth medium (GM) conditions. **B** Experimental and analysis workflow for rapid immunoprecipitation MS of endogenous proteins (RIME, *n* = 3 IPs from independent experiments per group). **C** Scheme depicting detected p53 peptides in samples immunoprecipitated with p53-specific antibody. *TAD* transactivation domain, *PRD* proline rich domain, *DBD* DNA binding domain, *HD* hinge domain, *OD* oligomerization domain, *RD *regulatory domain. **D** Scatter blot showing comparison of average spectral counts (avgSPC) in GM and SM samples for proteins precipitated with p53-specific antibody (DO1). Enrichment of proteins above *x* = *y* line indicates increased p53 interactions in SM vs GM samples. Inset shows proteins with higher avgSPC values. **E** p53 interaction network enriched under SM over GM from SAINT core analysis (cut off: SAINT probability SP ≥ 0.9). Encircled are proteins that are known p53 interactors (BioGRID, red) and/or mapped to the Reactome pathway “Metabolism of carbohydrates” (RSA-HAS-71387, green). **F** Dot blot showing results of SAINT score analysis of differential nuclear p53 interactors GM vs SM. Cut-offs SAINT probability (SP) ≥ 0.95; SP ≥ 0.8; SP < 0.8 indicated with grey scaled circles. Relative abundance represented by circle diameter. Blue tones indicate the respective average spectral counts (avgSPC). **G** Western blot showing immunoprecipitation after overexpression of HA-tagged p53 and/or FLAG-tagged UGP2 in HepG2 p53KO cells. Samples were treated with either GM or SM for 24 h and proteins precipitated with FLAG-beads. p53 co-eluted with UGP2 exclusively under SM conditions. **H** Western blot showing immunoprecipitation of overexpressed p53HA in HepG2 p53KO cells with co-eluted SORBS1 protein under GM and SM conditions. Samples were treated with either GM or SM for 24 h and proteins precipitated with anti-HA-beads

Our data identify novel p53 interaction partners during the immediate-early response to starvation and show that, as a consequence, p53 is stabilized in the nucleus. Hence, we next sought to investigate nuclear p53 interactions upon prolonged starvation. First evidence of starvation-mediated nuclear p53 interactions was derived from cross-linking HepG2 cells followed by Western blot with *α*-p53 antibody. This approach showed p53-containing high-molecular weight complexes in cells under growth medium (Fig. 4A). When incubated in starvation medium for 24 h—when p53 is mainly nuclear (Fig. 1D, E)—this complex formation increased in abundance (Fig. 4A). Importantly, this signal is p53-specific, as it was almost absent in p53KO cells (Fig. 4A). To further define the nuclear p53 interactome under starvation we employed rapid immunoprecipitation (IP) MS of endogenous proteins (RIME) [57]. For this, p53-proficient HepG2 cells were kept in either growth or starvation medium for 24 h, before they were cross-linked with formaldehyde. Nuclei were isolated, lysed, and chromatin was immunoprecipitated with p53 [with an IP-validated DO1 antibody (Fig S4A)] or with isotype-matched IgG control antibody. Enriched eluates were analyzed with LC-MS/MS (Fig. 4B). High Pearson correlation values between replicate p53 pull-downs (> 0.9; Fig S4B) and clear principal component (PCA) separation of p53-pull down groups from IgG controls (Fig S4C) indicates the robustness of the protocol. From the LC-MS/MS-identified 1008 proteins, stringent filtering against the respective IgG groups (SAINT score = 0.85) [58] yielded 82 p53 IP-specific proteins (Fig. 4B, Table S3). Validating efficient pull-down, six p53 peptides were detected in both p53-pulldown groups (but not in the IgG samples; Fig S4D) with an average sequence coverage of 16% (Fig. 4C). A lack of peptide detection in the *N*-terminus and the central DBD of p53 suggests that these domains were not amenable to trypsin digestion, likely because they were obscured by molecular interactions in the cross-linked material. The majority of the 82 p53 co-enriched proteins are differentially overabundant in the starvation medium group (Fig. 4D) confirming p53 nuclear translocation (Fig. 1D, E) and the increase in high molecular weight complexes upon 24 h of starvation (Fig. 4A). Depicting these interactors in a network, reveals a list of known (red edges in Fig. 4E) and novel potential p53 interactors, including several chromatin-related proteins, such as PPP1CC which has been previously shown to modify RNA polymerase II phosphorylation state [59], and mediator complex family member MED12. These interactions indicate that p53 is intricately involved in transcriptional complexes upon starvation and is conserved when using a more stringent SAINT score (top 22 with SAINT score ≥ 0.95 in Fig. 4F). Based on our prior work on p53 as a coordinator of the response to fasting [28, 60], we were intrigued by the number of metabolic enzymes mapping to the REACTOME pathway “carbohydrate metabolism” (circled green in Fig. 4E). In particular, UGP2, an enzyme involved in glycogen synthesis, raised our attention as we reported previously that hepatic p53 loss leads to deregulation of glycogen storage [28]. We observed that UGP2 peptides were absent in RIME growth medium samples, while abundantly present in starvation conditions (three out of three replicates). This observation was also reflected in overexpression experiments, where UGP2-FLAG overexpression was pronounced in starvation compared to growth medium conditions (Fig. 4G). Importantly, p53 was co-immunoprecipitated specifically under starvation upon UGP2-FLAG pull-down (Fig. 4G), confirming the RIME result by reverse pull-down. Furthermore, with Sorbin and SH3 domain-containing protein 1 (SORBS1) we confirmed another previously unreported p53 interactor (as judged by the prePPI prediction server http://honig.c2b2.columbia.edu/) [61, 62] as increasingly interacting with p53 under starvation conditions (Fig. 4H). SORBS1 has been reported to be involved in glucose metabolism via insulin receptor regulation [63]. These findings will spur further investigations to delineate the functional significance of the nuclear interaction of p53 and SORBS1 or UGP2, as well as other candidates identified by the RIME approach.

### p53-specific starvation-response transcriptional landscape

**Fig. 5:**
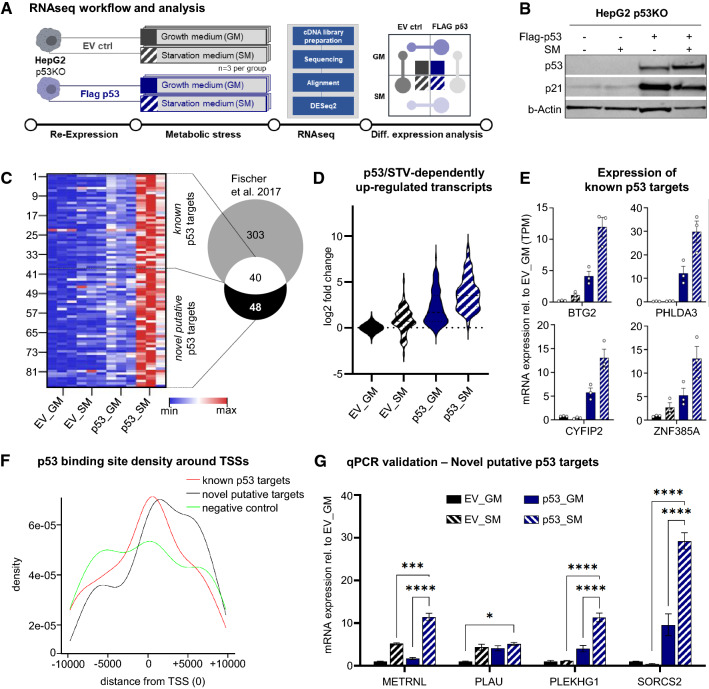
Transcriptome analysis reveals p53- and starvation-specific differentially expressed genes. **A** RNAseq experimental and analysis workflow in HepG2 p53KO cells re-expressing either FLAG-p53 or empty vector (EV) control (*n* = 3 per group). **B** Western blot showing FLAG-p53 re-expression and target gene expression (p21) in HepG2 p53KO cells treated with either growth medium (GM)or starvation medium (SM). Beta-actin as loading control. **C** Heat map showing 88 significantly differentially expressed gene transcripts (DESeq2, Wald-test with Benjamini–Hochberg correction, *p* < 0.05) enriched in p53 overexpressing cells and sensitive to starvation. Venn diagram showing overlap with p53 target gene meta-analysis (Fischer et al. 2017) indicating 48 novel, starvation-specific p53 target genes. **D** Violin blot showing fold-change (log2) distribution of 88 gene transcripts between treatment groups. EV_GM set to 1. **E** Exemplary expression profiles of known p53 target genes found in the RNAseq data set. EV_GM set to 1. **F** Density plot of p53 binding sites centered on transcription start sites (TSS) of gene sets of known, novel, and p53-unregulated (negative control) transcripts. **G** RT-qPCR validation of novel p53 target genes found in the RNAseq data set. Data are normalized to PPIA. Mean values ± SEM are shown and two-way ANOVA, Tukey’s multiple comparisons test was performed. ****p < 0.001, ***p < 0.005, *p < 0.05

Nuclear localization and interactions with chromatin regulators upon 24 h of starvation suggests that p53 functions to transactivate a starvation-specific set of target genes. Therefore, using our model with CRISPR/Cas9-mediated p53 knock out, we performed RNA-seq analysis with and without re-expression of full-length wild-type FLAG-tagged p53 both in growth medium and after prolonged starvation (workflow shown in Fig. 5A, validation of overexpression in Fig. 5B, group clustering in Fig S5A, and expression data in Table S4).

As p53 was previously suggested to solely act as transcriptional activator, while repressive actions were ascribed to secondary effects [20, 22], we limited our analysis to upregulated transcripts (min. two-fold change, adjusted *p*-value after BH correction < 0.05). Selecting transcripts that are specifically upregulated by p53 overexpression, as well as being starvation-responsive mainly in the presence of p53 (Fig. 5C and S5B), revealed a distinct set of 88 transcripts (Fig. 5C, D). Of these, 40 were known, validated direct p53 targets [20] and 48 are putative novel p53 targets under starvation conditions (Fig. 5C, Table S4). Expression patterns of *bona fide* p53 targets [20] are exemplified for the four transcripts *BTG2*, *PHLDA3*, *CYFIP2*, and *ZNF385A* in Fig. 5E. Confirming our approach and discerning known from potentially novel p53 target genes, mapping of the list of novel p53 targets to gene ontology biological processes or to REACTOME pathways did not yield any significant hits (FDR = 0.01). The list of known p53 target genes, on the other hand, returned several highly significantly enriched p53-specific terms and p53-related processes/pathways such as cell cycle control and DNA damage (Fig S5C). Next, we performed in silico mapping of p53 binding sites (consensus: GGACATGCCCGGGCATGTCY, Table S5) in the proximity of transcription start sites (TSSs) and compared TSS-centered p53 binding site distribution density from the 48 novel p53 target candidates with those of the 40 known transcripts and 48 transcripts that are starvation regulated in a p53-independent manner in our data set. This analysis showed that, similar to known targets, p53 binding sites in the identified putative targets are enriched around TSSs, whereas p53-unregulated transcripts showed no such enrichment peak (Fig. 5F and Table S5). Expression of four novel p53 target gene candidates with p53 binding sites within 1500 base pairs of their TSSs was confirmed via qPCR (Fig. 5G) and chromatin IP (ChIP)-qPCR confirmed enriched p53 binding in starvation over growth medium conditions for these four target genes (Fig S5D). Nutlin-3a treatment, however, led only to induction of two of these genes (METRNL and PLEKHG1, Fig S5E), while SORCS2 was even significantly decreased. This indicates that starvation-induced p53-dependent genes are regulated context-specific and that starvation and nutlin-treatment elicit different p53-mediated transactivation programs.

Taken together, our transcriptome analyses with p53 re-expression on a knock-out background show that p53 coordinates very specific transcriptome changes in response to starvation. Bioinformatics analyses suggest a number of novel starvation-specific p53 target genes, extending the current knowledge base of p53’s transactivation landscape.

## Discussion

The specifics of p53 action are highly dependent on the cellular context, the nature of the upstream stressor, and the intensity and duration of the stressor. Activated p53 signaling significantly influences cell fate decisions that range from survival to cell death. p53 activation through various nutrient deprivation conditions has been reported in several cancer model systems [64, 65]. Recent reports showed that p53 protein abundance is increased in cultured hepatocytes by starvation [28] and that p53 signaling is necessary for the physiological response to fasting in the mouse liver [28, 30]. Furthermore, our most recent data provide evidence for the requirement of p53 for the effects of fasting or starvation in enhancing liver cancer therapy [29]. Thus, our findings add to a flourish of recent reports that establish p53 as central and potent metabolic regulator in transformed as well as in non-transformed cells [49, 65]. Here, we focused on p53’s role as a starvation-response factor and aimed to systematically delineate the entire signaling cascade in response to a starvation stimulus. Using tailored omics methods combined with in-depth bioinformatics analyses and biochemical assays, we define the p53 interaction network leading to nuclear p53 stabilization, the chromatin interactions under prolonged starvation, and the corresponding p53 “targetome” at the transcriptional level.

The interaction of p53 and MDM2 is instrumental for determining cellular p53 levels. Our data suggest a dissociation of MDM2 and p53 as a main mechanism of p53 stabilization under starvation. Preferential co-IP of p53 and MDM2 under non-starved conditions corroborates this (Fig S1E). That the pull down of p53 did not lead to a similar co-precipitation of MDM2 (reverse co-IP, Fig S1E) is likely due to the fact that the used p53 antibody targets the MDM2-binding site, which might be obscured in p53/MDM2 dimers. However, MDM2 can be regulated by many posttranslational modifications [18]. Our approach, performing p53-BioID to identify cytoplasmatic proteins vicinal to p53 within two hours of nutrient withdrawal, identified increased association with FKBP3. FKBP3 was shown to interfere with the p53/MDM2 feed-back loop by directly binding to and inducing auto-ubiquitination and degradation of MDM2, subsequently leading to p53 stabilization [39]. Given our observations that MDM2 levels continuously decline during the course of starvation (Fig. 1A) and that the FKBP3/p53 association is increased early after nutrient withdrawal, FKBP3-mediated MDM2 degradation is a plausible mechanism to explain p53 stabilization under starvation. Moreover, we observed a starvation-mediated dissociation of p53 and several members of the 14-3-3 protein family in the BioID data set. From the seven known human 14-3-3 isoforms [41], our BioID data suggest p53 to associate with five, each showing high sequence coverage and each of which are enriched in growth medium when compared to starvation medium conditions. Dissociation from 14-3-3 proteins under starvation could therefore constitute a part of the mechanism of p53 stabilization and nuclear translocation, as all members of the 14-3-3 family were shown to directly or indirectly contribute to regulation of p53 activity [41] and a commonly described function of 14-3-3 proteins is sequestering proteins in the cytoplasm [66].

Further insights from the BioID-derived p53 interactome include the starvation-enriched association with the known interactor HGMB1 [67, 68] that was shown to prime p53 for DNA interaction [69], and an unexpectedly large number of metabolic enzymes that are preferentially enriched in growth medium conditions. For example, G6PD was previously described to directly interact with p53 to regulate glycolytic flow through the PPP, supporting biosynthetic processes during growth of HCT116 colon cancer cells [19]. Finding G6PD in our BioID data set validates our approach and suggests that among the other detected metabolic enzymes physical interactions with p53 are likely and may lead to respective functional consequences. In support of involvement of p53 with the PPP, we also found PRPS1 as a candidate p53 interactor that not only controls purine biosynthesis but also the PPP [70], therefore playing a central role in metabolic and oncogenic pathologies [71]. Along these lines, we also find association of p53 with two enzymes involved in oncometabolite production, IDH1 and PGHDH. A number of tumor entities harbor PHGDH overexpression or IDH1 mutations and the subsequent changes in cancer metabolism can be targeted [44]. Our screen also revealed major glycolysis pathway enzymes (PGK1, PKM, LDHA) as potential p53 interaction partners, raising the possibility that p53 is involved in regulation of glycolytic activity in nutrient-rich conditions via protein–protein interaction, while, upon nuclear translocation under starvation, p53 mainly seems to act as transcription factor. However, the BioID approach only informs about proximity throughout the labeling time window and does not provide evidence of direct binding. In the case of G6PD, direct p53 binding was shown to inhibit enzyme activity by prevention of an active G6PD dimer [19]. As this dimerization mechanism is very specific for G6PD activity, it might be intriguing to evaluate other potential effects of (direct or indirect) p53 interactions with the metabolic enzymes detected in our screen.

AMP-activated protein kinase (AMPK) is a central energy sensor with multiple outputs aimed to regenerate ATP levels when cellular energy levels run low [72]. In cancer cells, AMPK was shown to phosphorylate p53 to initiate a mitotic checkpoint upon glucose starvation [64]. The kinase PAK2 has been detected in an AMPK substrate screen and its phosphorylation at Ser20 was shown to increase with rising AMPK activation during nutrient deprivation [73]. Together with our novel finding of a direct p53/PAK2 interaction, which was found to decrease upon starvation, one could surmise that AMPK-mediated PAK2 phosphorylation releases p53 from the p53/PAK2 complex. Our data, functionally probing this axis through RNAi-mediated and pharmacological PAK2 inhibition, indicate small but consistent effects on the p53 signaling pathway that, at least to some degree, resemble starvation conditions. We note that nutrient withdrawal is a pleiotropic stimulus for cells that might elicit several signaling events impinging on p53, PAK2 signaling possibly being one of them. However, our data add another regulatory level to the nutrient sensing AMPK/p53 axis that warrants further investigation.

Further confirming p53 translocation under prolonged starvation, we found exclusive enrichment of potential p53 interactors in starvation medium over growth medium conditions (Fig. 4D) using RIME, a protocol that starts with nuclei isolation of cross-linked cells followed by MS identification of proteins co-immunoprecipitated with p53. Using IgG pull-down to control for background contamination [47] yielded a *bona fide* starvation-mediated, nuclear p53 interactome. Verifying the validity of this approach, we identified several p53 peptides mostly from its C-terminal domains (Fig. 4C). This suggests that the *N*-terminal domain and the DBD are obscured in chromatin complexes and therefore not amenable for tryptic digestion and that p53 may be largely bound to DNA. Further (structural) studies are needed to resolve the mode of p53/DNA interaction under starvation conditions. Our RIME approach also identified some known p53 interactors (e.g. MAPK1 [74, 75]) and a number of proteins not known to directly associate with p53. Among these, we focused on UGP2, which we confirmed by reverse co-IP (Fig. 4G). UGP2 is an essential enzyme that synthesizes UDP-glucose from glucose-1-phosphate as a precursor for glycogen synthesis [76, 77]. A plausible scenario is that nuclear translocation of p53 in starvation conditions facilitates UGP2 nuclear transport (or retention) to quench cytoplasmic glycogen synthesis under prolonged starvation. This would make glucose, derived from glycogenolysis or gluconeogenesis, available as an energy substrate during times of starvation, a mechanism that would promote both growth in cancer cells and effective energy partitioning during fasting in normal hepatocytes. Alternatively, a recent report described a surprising role of nuclear glycogen synthesis involving nuclear UGP2 in non-small cell lung cancer progression [78], which could also be affected by p53 under starvation. Another finding from our RIME screen we could confirm with co-IP experiments, was the association of p53 with SORBS1, that has been shown to be involved in insulin receptor signaling [63]. In the light of recent findings, showing nuclear translocation and transcriptional activity of the insulin receptor [79], it would be intriguing to scrutinize the role of p53 in this process under starvation. Such scenarios need to be further tested, but we view the UGP2/p53 and SORBS1/p53 association as examples of how biological relevant hypotheses can be generated from data obtained with our approaches.

Finally, we devised a tailored transcriptome study to delineate the p53-specific transcriptional response under starvation. CRISPR/Cas9-mediated knock out of p53 in our cell model followed by long-term starvation, yielded transcripts that are regulated by starvation in a p53-independent manner. Subtracting this set from starvation-responsive transcripts upon isogenic re-expression of full-length p53 delivers a starvation-specific targetome, containing known as well as potential novel p53 target genes. To further increase confidence in the putative novel targets, we used in silico binding site prediction and found an enrichment of p53 binding sites in the set of novel putative p53 target candidate genes when compared to transcripts that are starvation-regulated in a p53-independent manner. These putative targets have been confirmed to be increasingly p53 bound in starvation condition by ChIP-qPCR (Fig S5D) and two of the four investigated genes are also inducible by nutlin treatment (Fig S5E). That the other two genes are not upregulated by nutlin treatment, as upon starvation (Fig. 5G), indicates that starvation elicits a very specific p53 transactivation landscape in HepG2 cells.

**Fig. 6:**
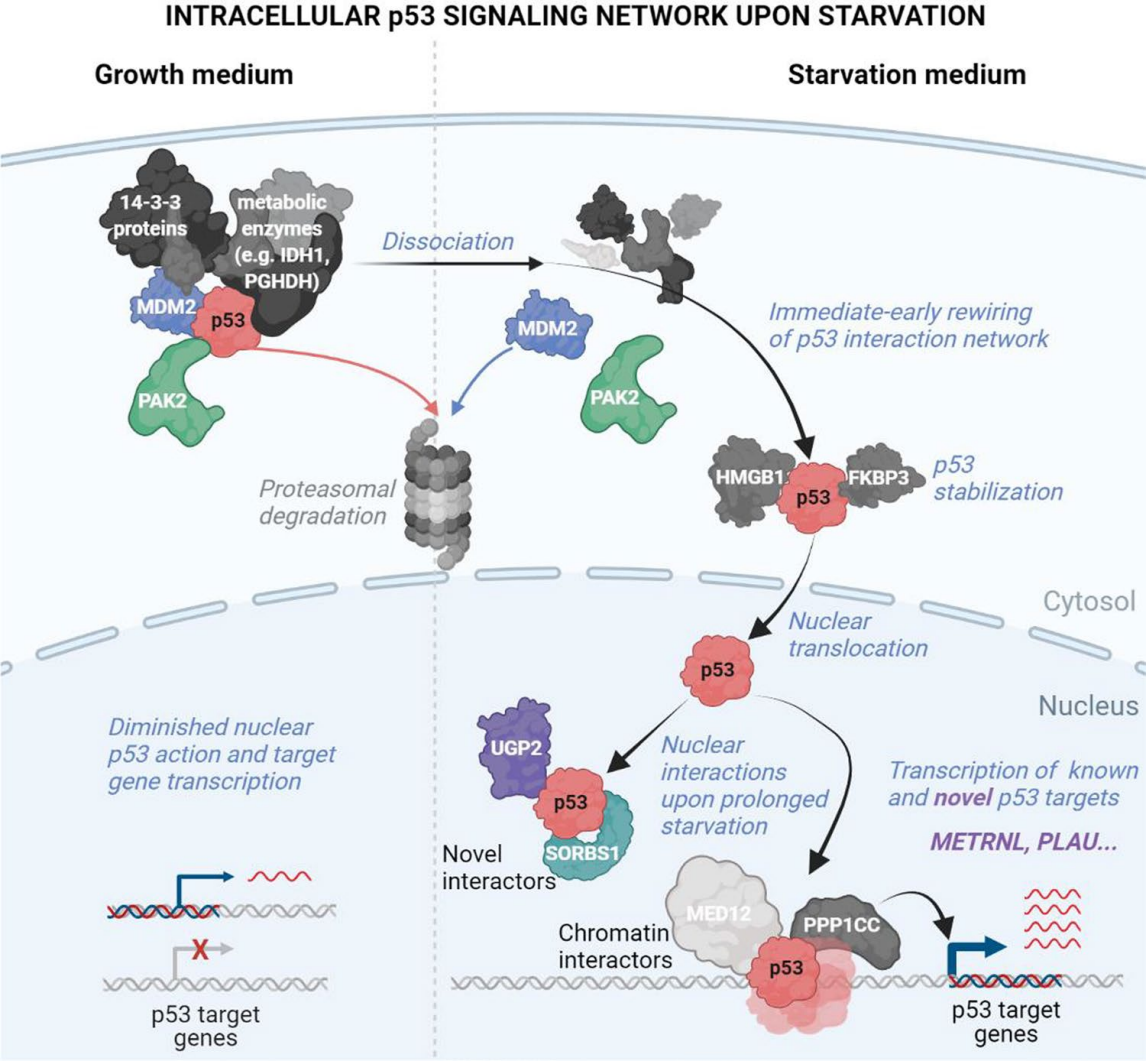
Model of p53 signaling and interactomes upon starvation. p53 signaling and interactomes upon starvation suggested by our complementary approaches. Processes investigated are in blue italics. Proteins that are individually investigated are colored. Grey scale proteins are examples derived from affinity purification MS and described in the text. Protein shapes are schematic with no relation to native conformations. Illustration was created with BioRender.com

Taken together, our study systematically dissects upstream mechanisms that lead to nuclear p53 stabilization upon starvation, as well as downstream interaction networks and the transcriptome response (Fig. 6). Verification experiments for selective findings prove the validity of the stringent experimental designs and bioinformatics analyses, and suggest novel starvation-specific mechanisms, such as PAK2-mediated p53 stabilization, nuclear interaction between p53 and UGP2 or SORBS1, and novel starvation-specific downstream target genes. These results are indicative for the utility of our complementary omics approaches to extend the already vast landscape of regulatory and functional p53 elements (summarized in Fig. 6).

Our study provides unprecedent details of the p53 signaling cascade upon nutrient withdrawal, thereby expanding the universe of p53 action and interaction in the context of cellular stress. However, we note that our approaches have a number of limitations. First, global approaches such as the ones used herein might produce a considerable number of indirect interactions (MS-based approaches) or indirect regulatory targets (transcriptomics), and may produce false-positive candidates. The latter problem is particularly pertinent to affinity-based proteomics methods that are known to enrich for unspecific proteins merely based on their over-abundance in the specific cellular context. To compensate for this complication, we spent considerable efforts to estimate background noise in the experimental contexts investigated. To this end, we used the same number of background control samples in both the BioID (i.e. EGFP fusion vector) and RIME (i.e. IgG pulldown) experiments and analyzed all our samples in triplicates. In addition, stringent cut-offs in the bioinformatics analyses using the SAINT score [47, 58] reduced the space of potential interactors and increased the degree of confidence in our final data sets. Finally, we note that our findings might be specific to HepG2 cells that are transformed and derived from juvenile hepatoma. For instance, the results with PAK2 inhibitor could not be reproduced in the hepatocellular carcinoma (HCC) cell line Huh6 or in HCC organoids, indicating that this pathway may be specifically relevant in starved HepG2 cells. However, the data presented herein might help to shed mechanistic detail on the therapy-sensitizing effects of fasting in dependence of the p53 status in HepG2 cells and in HepG2 xenografts, as recently published by our lab [29].

## Materials and methods

### Cell culture

Hepatoma-derived cells (HepG2) were purchased from ATCC (HB-8065). Cells were maintained in DMEM (gibco, 41966-029) containing 4.5 g/l glucose supplemented with 10% (v/v) heat inactivated fetal bovine serum (FBS; HyCloneTM, SV30160.03) and 1% Penicilin/Streptamycin (gibco, 15140-122) (“growth medium”, GM). Cells were cultured in a humidified atmosphere of 5% CO_2_, 95% air at 37 °C. From those cells, HepG2 p53 knockout cell lines (HepG2 p53KO) were generated using p53 CRISPR/Cas9 KO plasmid (h) (sc-416469, Santa Cruz Biotechnology). For starvation, GM was removed, cells were washed with phosphate buffered saline (PBS; gibco, 10010-015), and treated in HBSS (gibco, 14175-053) containing 1 g/l glucose supplemented with 10 mM HEPES (gibco, 1560-080) (“starvation medium”, SM). PAK2 gene silencing was conducted using Dharmacon™ ON-TARGETplus siRNAs (SMARTpool format) synthesized by Horizon (L-003597-00-0010). Non-targeting SMARTpool was used as control (Horizon, D-001810-10-05). siPAK2 or siCTRL SMARTpools were transfected in HepG2 wt cells with transfection reagent DharmaFECT 4 (Horizon, T-2004-02) at a concentration of 100 nM per well. After applying siRNA according to manufacturer’s protocol, GM was changed after 24 h of incubation and cells maintained for another 24 h in GM before harvesting in either RNA lysis buffer for qPCR or hypotonic buffer for subcellular fractionation.

### Compound treatments

For treatments with different small molecules, the following concentrations were used:

10 µM nutlin-3a (Biomol, Plymouth Meeting, PA, USA),

10 mM cycloheximide (CHX; Caymen Chem, 14126).

10 µM MG132 (Calbiochem, 474790).

20 nM to 1 µM FRAX597 (Selleckchem, S7271).

### Cell lysis

Lysis buffers were supplemented with 1 × protease inhibitor cocktail (PIC) (cOmplete Tablets EASYpack, Roche, 04693116001) and 1 × PhosSTOP (Roche, 04906837001). Total protein lysates were obtained after washing the cells with PBS, and application of the respective buffers provided in each protocol segment.

### Western blot (WB)

#### Whole cell lysates

Cultured cells were scraped and collected in radioimmunoprecipitation assay (RIPA) buffer (50 mM Tris–HCl, 150 mM NaCl, 2 mM EDTA, 50 mM NaF, 0.1% SDS, 0.5% Na-deoxycholate, 1% NP-40, adjusted to pH 7.2–7.4) supplemented with PhosStop and PIC. Samples were sonicated for 5 min (30 s on/off) in a cooled water bath ultrasound sonicator (Bioruptor, Diagenode). Cell lysates were centrifuged (14,500 rpm 15 min 4 °C) and clear supernatants were used to measure protein concentrations with a bicinchoninic acid assay (BCA; Thermo Fisher Scientific). Immunoblotting was performed as described elsewhere [28]﻿, using 50 µg protein of each sample.

#### Subcellular fractionation

Cytoplasmic and nuclear fractions were separated using a method previously described (Baldwin, Rockland). Briefly, cells were scraped in hypotonic cytoplasmic lysis buffer (10 mM HEPES, 60 mM KCl, 1 mM EDTA, 1 mM DTT, adjusted to pH 7.6) and incubated for 10 min on ice. After addition of IGEPAL (stock solution: 10%) to a final concentration of 0.5%, cells were vortexed for 15 s, incubated on ice for 1 min, vortexed again for 15 s and centrifuged for 4 min at 1500 rpm and 4 °C. The supernatant was collected as the cytoplasmic fraction. Crude nuclei pellet was washed once with cytoplasmic lysis buffer (without PIC/PhosSTOP) and resuspended in nuclear extraction buffer (usually one fifth of cytoplasmic buffer volume used; 20 mM Tris-HCl, 100 mM NaCl, 1.5 mM MgCl_2_, 0.2 mM EDTA, 0.05% SDS and 25% (v/v) glycerol, adjusted to pH 8.0). The nuclear fraction was sonicated for 5 min (30 s on/off) in a cooled water bath ultrasound sonicator (Bioruptor, Diagenode). After sonication, 100 U/ml benzonase (Chem Cruz, sc-202391) was added to the nuclear lysates and incubated on ice for 1–2 h, vortexing the mixture periodically (15 min) to resuspend the pellet. Digested sample was centrifuged at 14,500 rpm for 15 min and 4 °C to precipitate any remaining cell debris. The supernatant was used as the nuclear fraction.

WES digital western blot (Bio-techne, Proteinsimple, Minneapolis, Minnesota) was performed according to the manufacturer’s guidelines, using 10 µg protein of each sample.

### RNA isolation and reverse transcription

After cell lysis with RNA-Lysis-Puffer T (peqGOLD VWR, 12-TRK-88) RNA was isolated with PeqGOLD Total RNA Kit (C-Line) (peqGOLD VWR, 12-6634-02) according to the manufacturer’s protocol. Sample purity and concentrations were measured with NanoDrop^®^ ND-1000 (peqlab Biotechnologie GmbH). Reverse transcription was performed with High-Capacity cDNA Reverse Transcription Kit (Applied Biosystems by Thermo Fischer Scientific, 4368814). RT Puffer (10×), 0,8 µl dNTP (100 mM), 2 µl RT Random Primer (10×), nuclease free water and MultiScribe Reverse Transcriptase (50 U/µl) were added to defined RNA concentrations according to the manufacturer’s protocol. The thermo cycler program was set to 10 min at 25 °C, 120 min at 37 °C, 5 min at 85 °C and subsequent cooling at 4 °C. cDNA was diluted afterwards to a concentration of 1–20 ng/µl and stored at − 80 °C.

### Real time quantitative PCR (qPCR)

qPCR was performed in 96-well plate (BioRad, HSL9601) or 384 well plates (BioRad, HSP3805) with a cDNA concentration between 1 and 20 ng/µl. A master mix of Blue SybrGreen qPCR (Biozym, 331416) and cDNA (ratio 2:1; 96-well plate: 5 µl SybrGreen:2,5 µl cDNA or 384-well plate: 3µl SybrGreen:1,5 µl cDNA) were prepared and added to each well to pre-pipetted primer pairs (final concentration 80 nM per primer) to a final volume of 10 µl (96-well plate) or 6,5 µl (384-well plate) per well. After performing qPCR in a C1000TM Thermal Cycler (BioRad, CFX96 Real-Time System or CFX384 Real-time System) (Program: 1 cycle (10 min) at 95 °C; 40 cycles: 15 s at 95 °C, 1 min at 60 °C, 1 min at 72 °C; 1 cycle: 30 s at 95 °C, 30 s at 60 °C, 30 s at 95 °C) data was analyzed with Bio-Rad CFX Manager 3.1 software. The list of primers is provided in Table S6.

### Viability assay

For the viability assay, 2 × 10^5^ cells/well were seeded in a 96-well plate (Thermo Fisher Scientific) in 200 µl of GM to reach full confluency. Cells were allowed to attach for 24 h at 37 °C in 5% CO_2_, after which the medium was discarded, and cells were washed with PBS and treated with either GM or SM for 24 h. Cell viability was analyzed using an EZ4U assay (Biomedica Immunoassays) according to the manufacturer’s instructions. Briefly, at the end of treatment the media was replaced with fresh GM (200 μl/well) and 20 μl/well EZ4U working solution. After 2 h of incubation at 37 °C, the absorbance was measured at 492 nm with a reference-wavelength of 620 nm (Spark^®^ 10 M multimode microplate reader).

### Molecular cloning

Inserts (p53 wt sequence or EGFP wt sequence) for V5-p53-miniTurbo-NES and V5-EGFP-miniTurbo-NES were amplified from Flag-p53 (Addgene 10838; Primer: F: 5′- ACAGCGCTAGCGAGGAGCCGCAGTCAGATC-3′ R: 5′-ACAGCGCTAGCGGATCCGTCTGAGTCAGGCCCTTCTGT-3′) or EGFP (Addgene, 13031; Primer: F: 5′- ACAGCGCTAGCGTGAGCAAGGGCGAGGAG-3′ R: 5′-ACAGCGGCTAGCGGATCCCTTGTACAGCTCGTCCATGCCG AGAG-3′), cloned into the NheI site in V5-miniTurbo-NES (Addgene, 107170) and the correct sequence validated by Sanger sequencing.

### Proximity-biotinylation (BioID)

HepG2 p53KO cells were cultured in collagen-coated T75 flasks (approx. 1.5 × 10^7^ cells) and transiently transfected (VIAfect transfection reagent, E4981, Promega) with either V5-p53-miniTurbo-NES or V5-EGFP-miniTurbo-NES. After 24 h of transfection, intracellular biotinylation was induced by changing cell culture media to either GM or SM (medium composition described in subsection cell culture) both containing 250 µM biotin. Each group was analyzed in 3 biological replicates (GM_p53, SM_p53, GM_EGFP, SM_EGFP; 12 samples total). After 2 h of biotin labeling, samples were harvested and processed as previously published (Roux, 2018). In brief, cells were lysed in lysis buffer (8 M urea, 50 mM Tris-HCl, pH 7.4) containing protease inhibitor and 1 mM dithiothreitol (DTT). After addition of 20% Triton X-100 to a final concentration of 1% and sonication for 10 min (30 s on/off) in a cooled water bath ultrasound sonicator (Bioruptor, Diagenode), samples were subjected to affinity purification with streptavidin beads (Invitrogen, 65001) on a rotator o/n at 4 °C. Afterwards, beads with captured biotinylated proteins were thoroughly washed (10 times) with washing buffer (8 M urea, 50 mM Tris-HCl, pH 7.4) and two final washing steps with 50 mM Tris-HCl, pH 7.4 and 50 mM AMBIC containing 1 mM biotin to block remaining streptavidin on the beads. The beads including the captured proteins were reconstituted in 100 mM Tris-HCl, pH 8.5, 2% sodium deoxycholate (SDC) and reduced/alkylated with 5 mM TCEP/30 mM chloroacetamide at 56 °C for 10 min. After that, the proteins were quantified (using a BCA assay) and digested with 1:100 Lys-C and 1:50 trypsin overnight at 37 °C with continuous horizontal shaking. Digestion was stopped by adding 1% trifluoroacetic acid (TFA) to a final pH of two, in which SDC was completely precipitated. SDC and the beads were removed by centrifugation at 14,000 rpm for 10 min and the supernatant, containing peptides was desalted on an Oasis HLB plate (Waters). Peptides were dried and dissolved in 2% formic acid before LC-MS/MS analysis.

#### Mass spectrometry run

One thousand ng of each sample was analyzed using an Ultimate3000 high-performance liquid chromatography system (Thermo Fisher Scientific) coupled to an EXPLORIS 480 mass spectrometer (Thermo Fisher Scientific). Buffer A consisted of water acidified with 0.1% formic acid (FA), while Buffer B was 80% acetonitrile (ACN) and 20% water with 0.1% FA. The peptides were first trapped for 1 min at 30 µl/min with 100% Buffer A on a trap (0.3 × 5 mm with PepMap C18, 5 μm–100 Å Thermo Fisher Scientific); after the trapping peptides were separated by a 50 cm analytical column packed with C18 beads (Poroshell 120 EC-C18, 2.7 μm, Agilent Technologies). The gradient was 9–40% B in 40 min at 400 nL/min. Buffer B was then raised to 55% in 5 min and increased to 99% for the cleaning step. Peptides were ionized using a spray voltage of 2 kV and a capillary heated at 275 °C. The mass spectrometer was set to acquire full-scan MS spectra (350–1400 m/z) for a maximum injection time of 120 ms at a mass resolution of 60,000 and an automated gain control (AGC) target value of 300%. For a total cycle of 1 s the most intense precursor ions were selected for tandem MS (MS/MS). HCD fragmentation was performed in the HCD cell, with the readout in the Orbitrap mass analyzer at a resolution of 15,000 (isolation window of 1.4 Th) and an AGC target value of 200% with a maximum injection time of 25 ms and a normalized collision energy of 28%.

#### BioID data analysis

All raw files were analyzed by MaxQuant v1.6.17 software using the integrated Andromeda Search engine and searched against the Human UniProt Reference Proteome (October 2020 release with 75,088 protein sequences). MaxQuant was used with the standard parameters (“Label-Free Quantification”, “iBAQ” and “Match between runs” were selected with automatic values) with only the addition of Deamidation (N) as variable modification. Data analysis was performed with Perseus v1.6.14: proteins reported in the file “proteinGroups.txt” were filtered for reverse, potential contaminants and identified by site. For the quantitation, we used the iBAQ values calculated by MaxQuant and we kept only proteins found in at least two biological replicates in each group. At this point intensities were log10 transformed and missing values were imputed by Perseus with the automatic settings (width: 0.3, down shift: 1.8, mode: separately for each column) leading to 2500 proteins left for statistical analysis with ANOVA testing (Benjamini–Hochberg FDR 0.05), z-score (mean per row) and hierarchical clustering (distance: Euclidian). Clusters were selected if enriched over EGFP background control under both nutrient conditions. Volcano bots were generated with Perseus 1.4.16 using the setting *t*-test, GM_p53 vs SM_p53, FDR0.05. KEGG overrepresentation analysis was performed in webgestalt.org [80] functional database ‘pathway–KEGG’ against ‘genome protein-coding’ reference list with 272 high confidence p53 interactors originating from the hierarchical clustering analysis (3 clusters enriched over EGFP background, Fig. 2C). In a second MaxQuant screen with the annotated proteins from the initial search only, biotinylated peptides were identified, by adding the modification Biotin(K) = “C(10) H(14) N(2) O(2) S” (+ 226.077598) possible on any K or protein N-term as search criteria. The resulting biotinylated peptides were filtered for occurring only in the p53 samples (Table S2). Biotinylated peptides are added in the search output (PRIDE) with file name “Biotin (K)Sites.txt” together with the other submitted files. Unique peptides found only in p53 samples were analyzed in Perseus v1.6.14: peptides from “peptideGroups.txt” were first filtered for reverse, potential contaminant, identified by site and afterwards only for occurring in p53 samples (Table S2). For SAINTscore analysis, the peptide counts for each sample were retrieved from MaxQuant output file “proteinGroups.txt” and prepared as input file for analysis in the Contaminant Repository for Affinity Purification (CRAPome). Analysis in CRAPome was conducted with experiment type: Proximity Dependent Biotinylation; Quantitation type: SPC without selecting CRAPome internal controls, instead only experimental controls as background (GM_p53, SM_p53, and CONTROL) EGFP control groups were merged to a single group, as we specifically look for proteins enriched over background control in both conditions (GM and SM), similar to the hierarchical clustering analysis. Interaction scoring was conducted with standard analysis options. For generating dot plots, the CRAPome output file was downloaded via the External Tools section and used as input file in ProHits-viz [48].

### Fluorescence microscopy

Cells were seeded onto collagen-coated glass Chamber Slides and transfected with ViaFECT (Promega) according to the manufacturer’s protocol. After 18 h, cells were washed two times with PBS and fixed with 1% paraformaldehyde (PFA; Thermo scientific) for 15 min at room temperature. PFA was removed and cells were washed two times with PBS. After washing, cells were blocked using Ultravision Hydrogen Peroxide Block (Thermo scientific, Rockford, USA) for 5 min at RT. Then, cells were incubated with primary antibody (p53 DO1, Santa Cruz, sc-126) diluted in Antibody diluent (Dako, California, USA) for 45 min in the dark. Cells were then washed two times with PBS, followed by incubation with secondary antibody Anti-rabbit IgG (H + L) Alexa Fluor 555 (Cell Signaling) for 30 min in the dark. Secondary antibodies were diluted 1:1000 in Antibody diluent and slides washed another two times with PBS after secondary antibody. Cells transfected with GFP-p53 (Addgene, 12091) overexpression vector were treated with MG132 for 2 h prior fixation and directly processed for fluorescence imaging without using primary or secondary antibodies. In both protocols, nuclei were counter-stained using DAPI 1:2000 (Thermo scientific) diluted in PBS for 5 min at RT. Finally, chambers were removed from the slides and the slides were washed twice with PBS before mounting with ProLongTM Gold antifade reagent (Invitrogen) and applying a cover slip. Slides were stored in the dark until visualizing on a PALM Microbeam Laser Microdissection microscope (Zeiss, New York, USA) using 40 × magnification. Images were analyzed using Zen software 2.3. Brightness and contrast were modified for enhanced visualization.

### Expression and purification of recombinant proteins

Expression constructs for the fragments of p53 (Uniprot ID P04637) corresponding to amino acid 1–94 (p53 TAD) and 94–312 (p53 DBD), as well as for PAK2 (Uniprot ID Q13177) corresponding to amino acid 1–212 were generated by synthesis of the corresponding optimized cDNA-constructs of p53 or PAK2 respectively and insertion of these cDNA into a pETM11-ZZ-His_6_ vector via NcoI/BamHI restriction digest.

For expression of recombinant unlabeled or 15 N labelled ZZ-His_6_ proteins, the bacterial expression vectors were transformed into Escherichia Coli BL21-DE3 Star strain cells. Cells were either grown in lysogeny broth medium for unlabeled proteins or minimum medium supplemented with 6 g ^12^C_6_H_12_O_6_ and 1 g ^15^NH_4_Cl at 37 °C until they reached an OD600 of 0.8, when protein expression was induced by addition of 0.5 mM IPTG. After proteins were expressed at 20 °C for 16 h, cells expressing disordered fragments (p53 TAD and PAK2 ^1–212^) were harvested in denaturing buffer (50 mM Tris–HCl pH 7.5, 150 mM NaCl, 2 mM Imidazol, 6 M Urea) and cells expressing folded fragments (p53 DBD) were harvested in non-denaturing harvesting buffer (50 mM Tris–HCl pH 7.5, 150 mM NaCl, 2 mM Imidazol, 2 mM tris(2-carbocyethyl)phosphine)), followed by sonication and centrifugation at 6198 rcf for 45 min. Proteins were purified from the lysate using Ni–NTA agarose (Qiagen) and the ZZ-His_6_ tag was cleaved by 2% (w/w) His_6_-tagged TEV protease treatment for 16 h at 4 °C. After a desalting step (HiPrep 26/10, GE Healthcare) on an ÄKTA Pure system (GE Healthcare) into a low imidazole buffer (50 mM Tris-HCl pH 7.5, 150 mM NaCl, 2 mM Imidazol), the cleaved protein was separated from the uncleaved protein and the His_6_ tagged TEV protease by Ni-NTA affinity chromatography. Finally, the proteins were purified using size exclusion chromatography (Superdex 75 Increase, GE Healthcare).

### Nuclear magnetic resonance (NMR) binding assay

For binding studies all proteins were equilibrated in the same buffer containing 20 mM Hepes pH 7.0, 50 mM NaCl, 2 mM TCEP. Samples for NMR measurements contained 100 µM ^15^N labelled p53 DBD in presence of 0 µM, 50 µM, 100 µM or 200 µM unlabeled PAK2^1−212^ and 10% D_2_O. For titrations using ^15^N labelled PAK2^1−212^ 50 µM were used in presence 0 µM, 50 µM and 100 µM p53 DBD and 10% D_2_O. ^1^H^15^N HSQC spectra of the aforementioned samples were recorded at 25 °C on a Bruker 600 MHz Avance Neo NMR spectrometer equipped with a TXI room temperature probe.

### Rapid immunoprecipitation mass spectrometry of endogenous proteins (RIME) for analysis chromatin complexes

HepG2 wt cells were grown to 100% confluency and treated with either SM or GM for 24 h. After crosslinking with 1% FA (prepared in either SM or serum–free GM) for 8 min, the reaction was quenched with glycine at a final concentration of 0.1 M and cells washed within the flask twice with ice-cold PBS. Cells were immediately frozen at − 80 °C and stored in flasks until use. Cell lysates were prepared as previously described [57] and nuclei extracted for immune-precipitation. IP was conducted with either DO1X (Santa Cruz, sc-126X) antibody for p53-specific pulldown or IgG2A isotype control (Cell Signaling, 61656). After thorough washing of the bead-bound proteins with AMBIC, proteins were digested on-bead with trypsin (1:50) o/n and for an additional 4 h on the next day. Derived peptides were desalted with C18 ultra micro spin columns and analyzed with LC–MS/MS.

#### Mass spectrometry run

One-tenth of each sample was measured by nano-HPLC (Dionex Ultimate 3000) equipped with an Aurora Series Emitter nanocolumn with CSI fitting (C18, 1.6 µm, 120 Å, 250 × 0.075 mm) (IonOpticks, Melbourne, Australia). Separation was carried out at 50 °C at a flow rate of 300 nl/min using the following gradient. Solvent A is 0.1% formic acid in water and solvent B is acetonitrile containing 0.1% formic acid: 0–18 min: 2% B; 18–100 min: 2–25% B; 100–107 min: 25–35% B, 107–108 min: 35–95% B; 108–118 min: 95% B, 118–118 min: 95–2% B; 118–133 min: 2% B. The Bruker maXis II ETD mass spectrometer was operated with the captive source in positive mode with following settings: mass range: 150–2200 m/z, 4 Hz, precursor acquisition control top20 (CID), capillary 1600 V, dry gas flow 3 L/min with 150 °C, nanoBooster 0.2 bar.

#### RIME data analysis

The MS/MS data were analyzed for protein identification and label-free quantification using MaxQuant 1.6.1.0 against the public database Swiss-Prot with taxonomy *Homo sapiens* and common contaminants (downloaded on 16.04.2019, 20482 sequences). Detailed search criteria were used as follows: trypsin, max. missed cleavage sites: two; oxidation on Met as variable modification; search mode: MS/MS ion search with decoy database search included; precursor mass tolerance ± 0.006 Da; product mass tolerance ± 80 ppm; acceptance parameters for identification: 1% PSM FDR; 1% protein FDR and 1% site decoy fraction. In addition, a label free quantitation and iBAQ values including the match between runs feature of MaxQuant was performed [81] requiring a minimum of two ratio counts of quantified razor and unique peptides while omitting the normalization step. Data processing was performed using Perseus software version 1.6.6.0, contaminants and reverse proteins created during database search were removed. Intensities were log10 transformed in order to lower the effect of the outlier values, filtered for three valid values in either GM_p53 or SM_p53, or at least four valid values in total, allowed no values in the IgG control groups. Missing intensities were replaced with random values taken from the Gaussian distribution of values using default parameters (width: 0.3, down shift: 1.8, mode: separately for each column), in order to simulate a value for low abundant proteins. Two-sample *t*-tests and volcano blotting were used to identify altered proteins between GM and SM conditions. PCA analysis and multi-scatter plot were generated in Perseus. For SAINTscore analysis, the peptide counts for each sample were retrieved from MaxQuant output file “proteinGroups.txt” and prepared as input file for analysis in the Contaminant Repository for Affinity Purification (CRAPome, lit). Analysis in CRAPome was conducted with experiment type: Endogenous pull-down; Quantitation type: SPC without selecting CRAPome internal controls, instead only experimental controls as background (GM_p53, SM_p53, and IgG CONTROL) IgG control groups were merged to a single group, as we specifically look for proteins enriched over background control in both conditions (GM and SM). Interaction scoring was conducted with standard analysis options. For generating dot plots and comparison of average spectral counts, the CRAPome output file was downloaded via the External Tools section and used as input file in ProHits-viz [48].

### Co-immunoprecipitation (IP)

HA-p53 (Genscript, OHu20059C) and/or UGP2-FLAG (Genscript, OHu02074D) were transiently overexpressed in HepG2 p53KO cells with ViaFECT (Promega) according to the manufacturer’s protocol. After 24 h of transfection, cell culture media was changed to either GM or SM and kept under those conditions for another 24 h. Next, cells were harvested by washing with ice-cold PBS and lysed in Co-IP buffer (25 mM Tris-HCl, 150 mM NaCl, 1 mM EDTA, 1% IGEPAL, 5% glycerol; adjusted to pH 7.4) containing PIC and PhosSTOP. After 30 min incubation on ice (passive lysis), the suspensions were sonicated for 5 min (30 s on/off) in a cooled water bath ultrasound sonicator (Bioruptor, Diagenode) at 4 °C. The lysates were centrifuged for 5 min at 14,500 rpm and 4 °C. Protein concentration was determined with a BCA protein assay kit. 500 µg of protein lysates was normalized to equal volumes with Co-IP buffer and used for precipitation. 30 µl Flag-beads slurry (Sigma, M8823) was used per mg protein, equilibrated in 500 µl Co-IP buffer for three times and incubated with the protein lysates over night at 4 °C with reciprocal shaking (BioSan MultiBio RS-24). Next day, flow-through was collected and used for control blots. Beads were washed three times with Co-IP buffer and proteins eluted from the beads with elution buffer (100 mM glycine, pH 3) at room temperature for 5 min.

### RNA-seq experiments and data analysis

Three SEQ libraries (TruSeq^®^ Stranded mRNA Library Prep) each of vehicle control (pcDNA4/HisMax, Invitrogen) and FLAG-p53 (pcDNA3 FLAG-p53, Addgene) re-expressing cells (electroporation) were prepared from purified mRNAs of HepG2 p53KO cells under untreated (GM) or treated (SM) conditions for a total of 4 × 3 (12) libraries. Raw sequencing data was generated on an Illumina NextSeq 550 and mapped to the human genome (hg18) using STAR alignment [82]. Differentially expressed genes were found using DEseq2 package in R, data were transformed (VST), Wald-Test performed on individual genes, and Benjamini–Hochberg corrected for multiple testing. Genes with an adjusted *p*-value ≤ 0.05 were used for further analysis.

### p53 motif analysis and motif density

Analysis was conducted using HOMER (http://homer.ucsd.edu/homer/index.html) [83]. A motif file was generated based on p53 consensus sequence “GGACATGCCCGGGCATGTCY” (Human, hg18) derived from MotifMap (http://motifmap.ics.uci.edu/) [84] using HOMER’s seq2profile.pl, allowing six mismatches (Table S5). Motif sites were identified via HOMER’s annotatePeaks.pl module in “TSS mode”, searching ± 10 kb around each gene’s TSS on either the sense or the antisense strand in the provided lists of genes of interest. A Gaussian density plot was generated in R (https://R-project.org/) (R Core Team, 2017) using the ggplot2 package (https://ggplot2.tidyverse.org) [85].

### ChIP-qPCR

ChIP-qPCR was performed according to an established protocol [28]. Briefly, HepG2 wt cells kept under GM or SM were cross-linked with 1% formaldehyde (Thermo Fisher Scientific) for 15 min at RT. Crosslinking was stopped by adding 125 mM glycine (final concentration) to the medium for 5 min. Cells were dounce homogenized with 40 strokes and chromatin was sonicated in Bioruptor^®^ Pico Microtubes (Diogenode, C30010016) for 10 cycles (30 s on/ 30 s off) using the Diagenode Bioruptor (Diagenode, B01020001). Fragment size was analyzed by agarose gel electrophoresis. IP was performed using precleared Protein G DynaBeads magnetic beads (Thermo Fisher Scientific, 10003D). 5 µg of the following antibodies was used: p53 (DO1X, Santa Cruz, sc-126X), IgG_2A_ (Cell Signaling, 61656). Immunoprecipitated chromatin and input chromatin were reverse cross-linked and column purified. DNA was subjected to SYBR green qPCR. Primers designed to span loci with p53 consensus sequences are listed in Table S6_oligos. Primers spanning loci without p53 consensus sequence served as negative control.

### Quantification and statistical analysis

Quantification and statistical analyses are given in the respective methods description.

### Supplementary Information

Below is the link to the electronic supplementary material.

Supplementary file1 (PDF 1068 KB)

Supplementary file2 (XLSX 971 KB)

Supplementary file3 (XLSX 138 KB)

Supplementary file4 (XLSX 198 KB)

Supplementary file5 (XLSX 18420 KB)

Supplementary file6 (XLSX 40 KB)

Supplementary file7 (XLSX 12 KB)

## Data Availability

Software: no customized software was used. Data resources: the BioID data obtained in this study has been uploaded to ProteomeXchange Consortium via the PRIDE partner repository with the dataset identifier PXD027734. The RIME data obtained in this study has been uploaded to ProteomeXchange Consortium via the PRIDE partner repository with the dataset identifier PXD028341. The RNA-Seq data obtained in this study has been uploaded to NCBI GEO datasets, under accession number GSE183127.
