# Supplementary material for: Complementary omics strategies to dissect p53 signaling networks under nutrient stress
Source: Cell Mol Life Sci. 2022 May 30;79(6):326. doi: 10.1007/s00018-022-04345-8 (PMC9151573; doi:10.1007/s00018-022-04345-8)
Supplement: Supplementary file 1 — Supplementary file1 (PDF 1068 KB) [file 18_2022_4345_MOESM1_ESM.pdf]

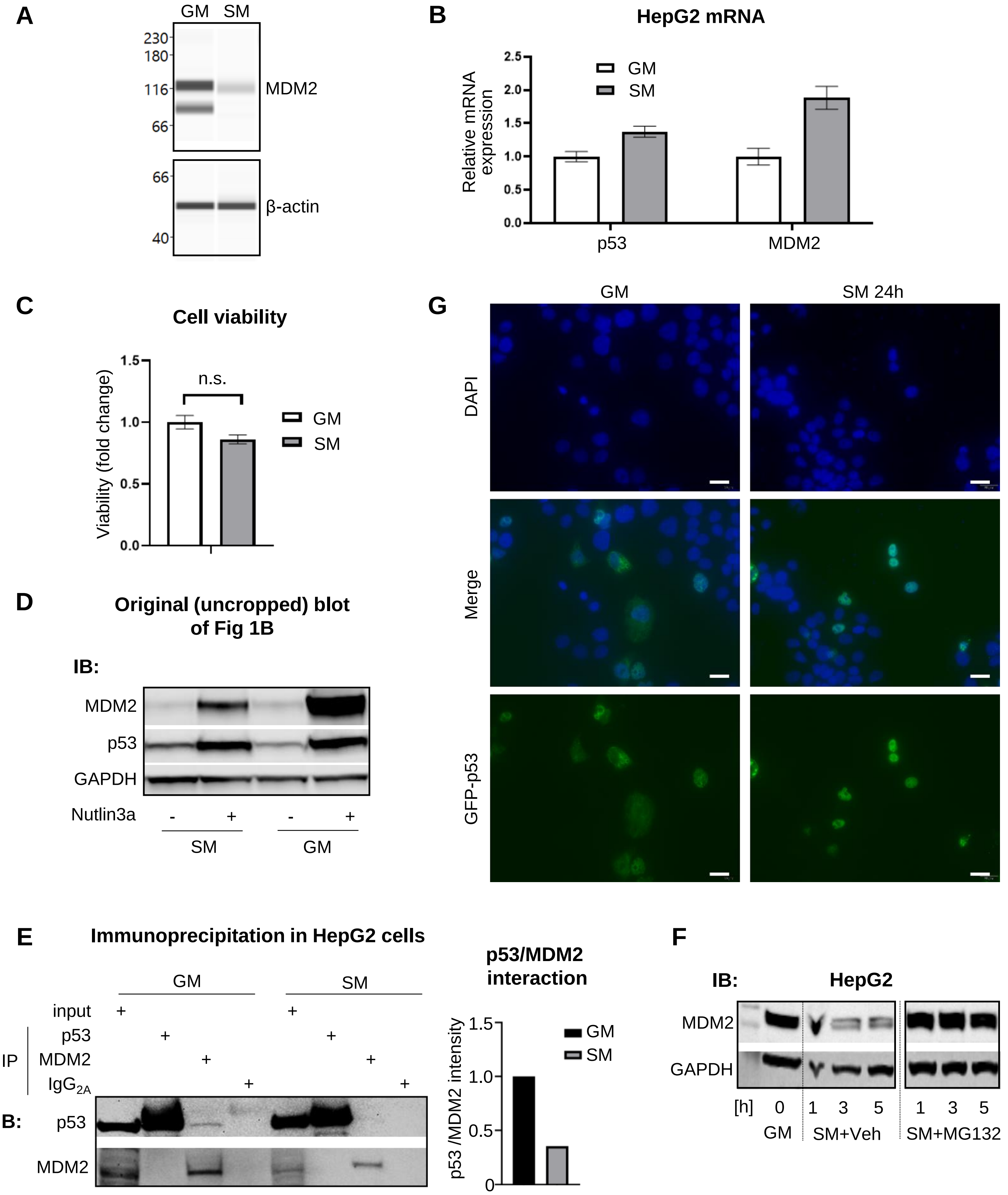

Supplemental Figure S1.

(A) Digital western blot (SimpleProt Wes system) of HepG2 cells in growth (GM) or 24 h starvation medium (SM) proved with anti-MDM2 antibody showing both the long and short isoform and b-actin as loading control.
(B) RT-qPCR fold change of p53, MDM2, mRNA expression levels in HepG2 cells determined with qPCR after 24 hours growth medium or starvation medium. Shown are replicate measurements of one representative out of three independent experiments. Data is shown as mean ± SEM.
(C) HepG2 cell viability after 24 hours growth medium or starvation medium determined with EZ4U assay. (n=4, Student's t test)
(D) Western blot showing regulation of p53, MDM2 after 24 hours growth medium or starvation medium containing pharmacological inhibitor nutlin-3a (10 µM). Uncropped blot from Figure 1B. GAPDH as loading control.
(E) Immunoprecipitation in HepG2 cells. Pull-down with p53, MDM2, and unspecific IgG antibody. p53 co-IPs with MDM2 preferentially in GM (quantified by dividing p53 by MDM2 band intensity (left panel)).
(F) Western blot of time course experiment in HepG2 cells showing MDM2 protein abundances after 1, 3, and 5 hours of starvation with either vehicle control or proteasomal inhibitor MG132. Panels are from same membrane with identical exposure. GAPDH as loading control.
(G) Immunofluorescence microscopy in GM and SM HepG2 cells after transfection with a GFP/p53-fusion vector and 2 h of treatment with the proteasomal inhibitor MG132 to stabilize proteins. DAPI is used to stain nuclei. Scale bars are 20 µm.

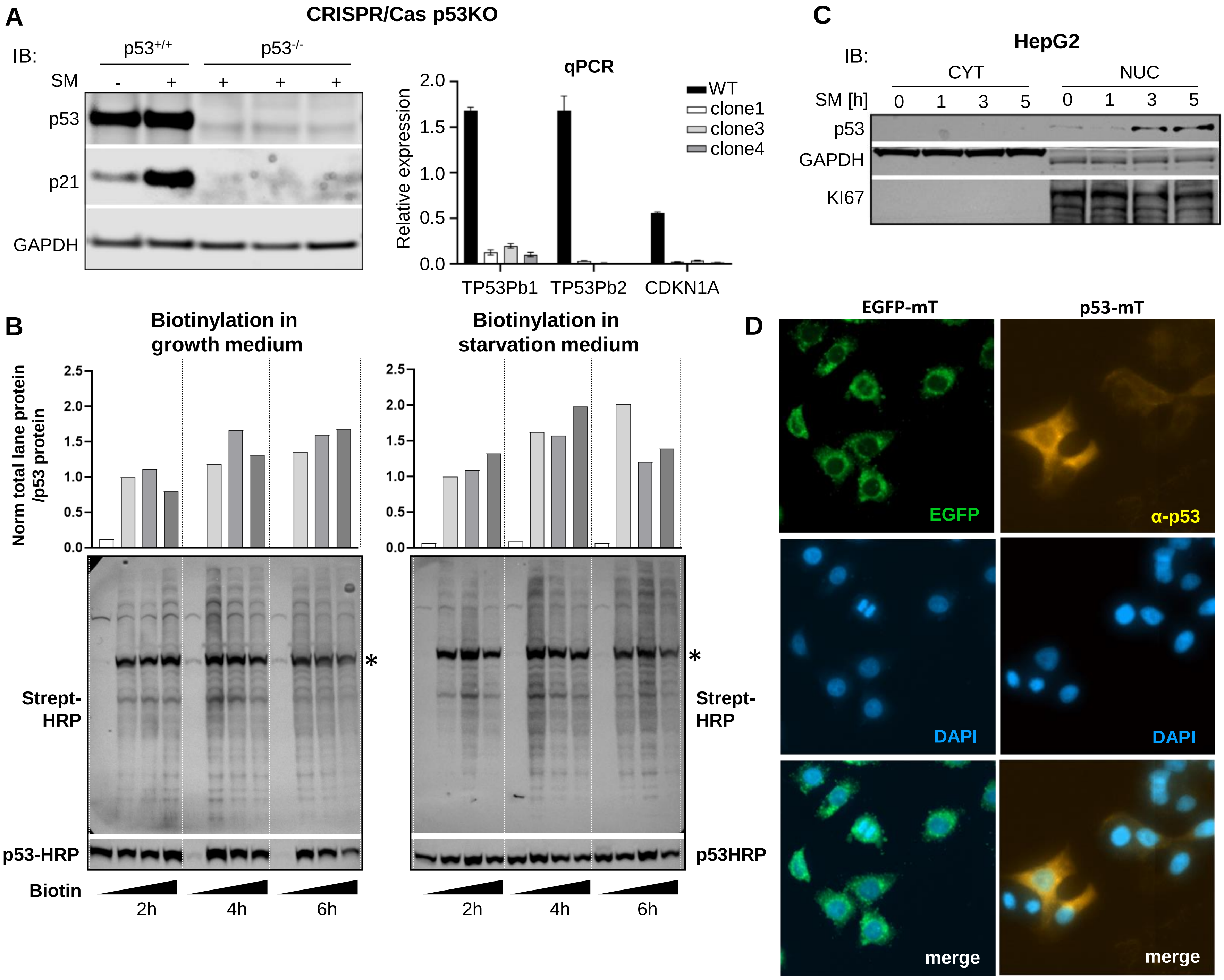

Supplemental Figure S2.

(**A**) Western blot and qPCR analysis showing successful CRISPR/Cas9-mediated knockout in three different clones. GAPDH as loading control. Pb1…primer pair 1, Pb2…primer pair 2, targeting different regions of p53 mRNA. CDKN1A…gene name for p21.
(**B**) Western blot detecting biotinylation with Streptavidin conjugated to HRP with samples from p53-mT overexpressing HepG2 cells with three different biotin concentrations (0 µM, 125 µM, 250 µM, 500 µM) in either growth medium or starvation medium cells for 2, 4, and 6 hours. Total lane protein was normalized to p53 overexpression values detected with p53-HRP antibody.
(**C**) Western blot of time course experiment in wild-type HepG2 cells subjected to 5 hours SM and subsequent subcellular fractionation. p53 antibody indicates nuclear p53 accumulation starting between 1 and 3 hours of treatment. GAPDH, KI67 as loading controls.
(**D**) Immunofluorescence showing cytoplasmic enrichment upon p53-mT and EGFP-mT overexpression. Nuclei are counterstained with DAPI.
(**E**) Hierarchical clustering. Unfiltered raw output from after Perseus ANOVA testing resulting in 609 significant proteins. Clusters shown in Fig 2C are marked green.
(**F**) Volcano blot showing total protein abundance changes between GM and SM in HepG2 p53KO un-transfected control cells.
(**G**) Volcano blot of 272 proteins enriched over EGFP background control. Enzyme mapping of EC numbers shown in blue for significantly changed (Fisher exact test, p=0.01), orange for not significantly changed proteins between GM and SM.

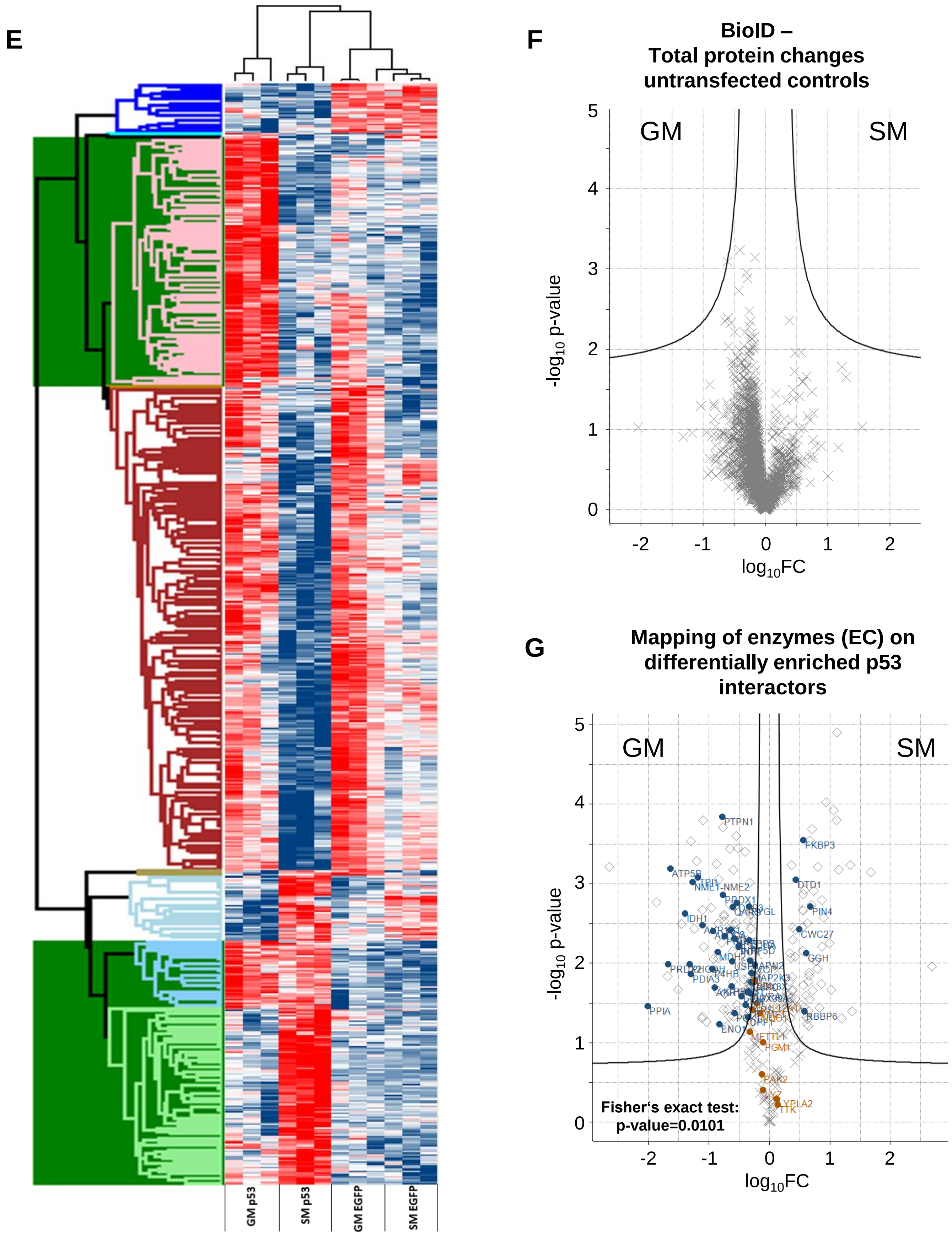

Supplemental Figure S2. (cont.)
(legend on previous page)

A

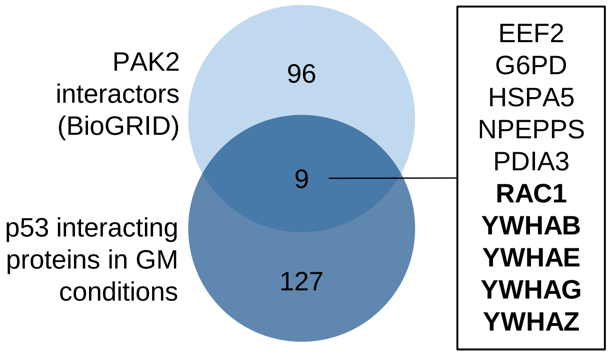

B Prediced 14-3-3 binding sites in PAK2 and TP53

>sp|**Q13177**|**PAK2_HUMAN**
Serine/threonine-protein kinase PAK 2 OS=Homo sapiens OX=9606 GN=PAK2 PE=1 SV=3

MSDNGELEDKPPAPPVRMSSTIFSTGGKDPLSANHSLKPLPSVPEEKKPRHKIISIFSGTEKGSKKKEKERPEISPPSDFEHTI
HVGFDAVTGEFTGMPEQWARLLQTSNITKLEQKKNPQAVLDVLKFYDSNTVKQKYLSFTPPEKDGFPSGTPALNAKGTEA
PAVVTEEEDDDEETAPPVIAPRPDHTKSIYTRSVIDPVPAPVGDSHVDGAAKSLDKQKKKTKMTDEEIMEKLRTIVSIGDPK
KKYTRYEKIGQGASGTVFTATDVALGQEVAIKQINLQKQPKKELIINEILVMKELKNPNIVNFLDSYLVGDELFVVMEYLAG
GSLTDVVTETCMDEAQIAAVCRECLQALEFLHANQVIHRDIKSDNVLLGMEGSVKLTDFGFCAQITPEQSKRSTMVGTPY
WMAPEVVTRKAYGPKVDIWSLGIMAIEMVEGEPPYLNENPLRALYLIATNGTPELQNPEKLSPIFRDFLNRCLEMDVEKR
GSAKELLQHPFLKLAKPLSSLTPLIMAAKEAMKSNR

Position	Peptide [-6:4]	ANN	PSSM	SVM	Consensus	pSer/Thr
36	PLSANH**S**LKPL	0.563	0.214	0.164	0.314	-
143	QKYLSF**T**PPEK	0.664	0.463	0.338	**0.488**	-
225	DKQKKK**T**KMTD	0.656	0.155	0.075	0.295	-
241	KLRTIV**S**IGDP	0.67	0.27	0.177	0.373	-
371	IHRDIK**S**DNVL	0.644	0.407	0.035	0.362	-
428	PKVDIW**S**LGIM	0.677	0.168	0.191	0.345	-

>sp|**P04637**|**P53_HUMAN**
Cellular tumor antigen p53 OS=Homo sapiens OX=9606 GN=TP53 PE=1 SV=4

MEEPQSDPSVEPPLSQETFSDLWKLLPENNVLSPLPSQAMDDLMLSPDDIEQWFTEDPGPDEAPRMPEAAPPVAPAP
AAPTPAAPAPAPSWPLSSSVPSQKTYQGSYGFRLGFLHSGTAKSVTCTYSPALNKMFCQLAKTCPVQLWVDSTPPPGTR
VRAMAIYKQSQHMTEVVRRCPHHERCSDSDGLAPPQHLIRVEGNLRVEYLDDRNTFRHSVVVPYEPPEVGSDCTTIHYN
YMCNSSCGGMNRRPILTIITLEDSSGNLLGRNSFEVRVCACPGRDRRTEEENLRKKGEPHHELPPGSTKRALPNNTSSSP
QPKKKPLDGEYFTLQIRGRERFEMFRELNEALELKDAQAGKEPG GSRAHSSHLKSKKGQSTSRHKKLMFKTEGPDSD

Position	Peptide [-6:4]	ANN	PSSM	SVM	Consensus	pSer/Thr
123	GTAKSV**T**CTYS	0.748	0.274	-0.008	0.338	-
140	FCQLAK**T**CPVQ	0.831	0.85	0.455	**0.712**	-
185	HERCSD**S**DGLA	0.724	0.461	0.057	0.414	-
269	NLLGRN**S**FEVR	0.621	0.37	0.123	0.371	-
284	PGRDRR**T**EEEN	0.616	0.473	-0.187	0.301	-
366	GGSRAH**S**SHLK	0.815	0.992	0.614	**0.807**	-
387	KKLMFK**T**EGPD	0.588	0.104	0.046	0.246	-

Highly scored by three methods
Highly scored by two methods only
Highly scored by one method only

ANN - Artificial Neural Network (cut-off = 0.55)
PSSM - Position-Specific Scoring Matrix (cut-off = 0.80)
SVM - Support Vector Machine (cut-off = 0.25)
Consensus - Average of the scores provided by the three methods (cut-off = 0.50)
pSer/Thr sites are obtained from UniProt and PhosphoSitePlus

C NMR binding assay: titration of PAK2$^{1\text{-}212}$ to p53 DBD

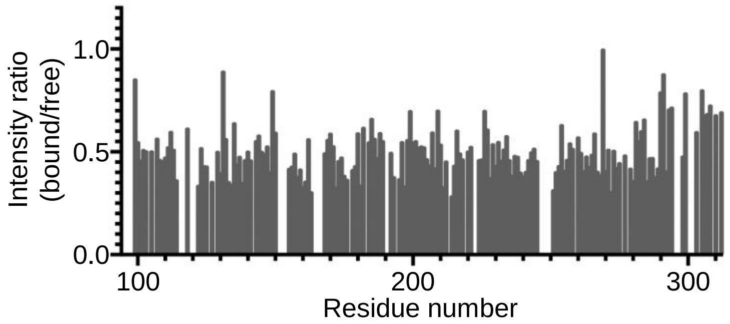

D NMR spectrum p53-TAD and PAK2 1-212

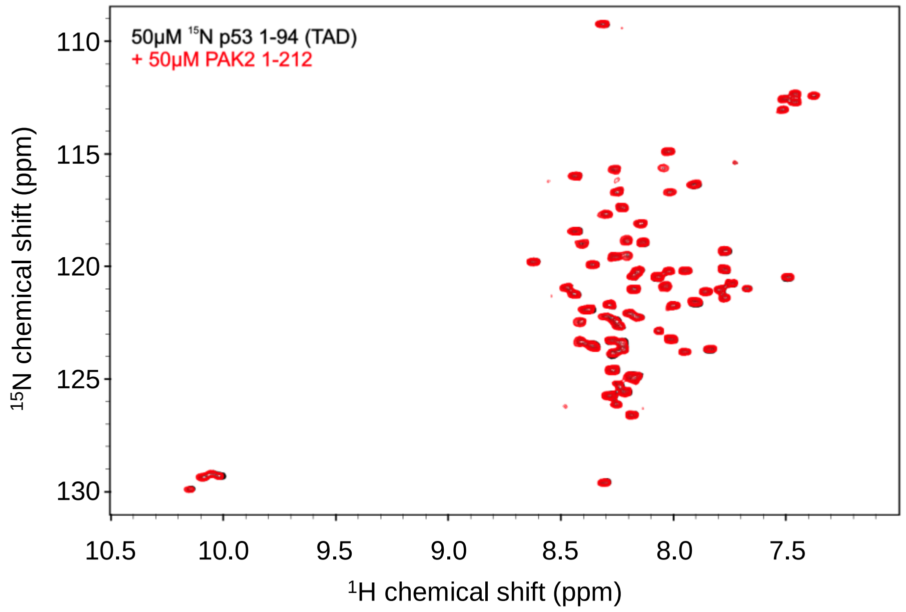

E Proposed model

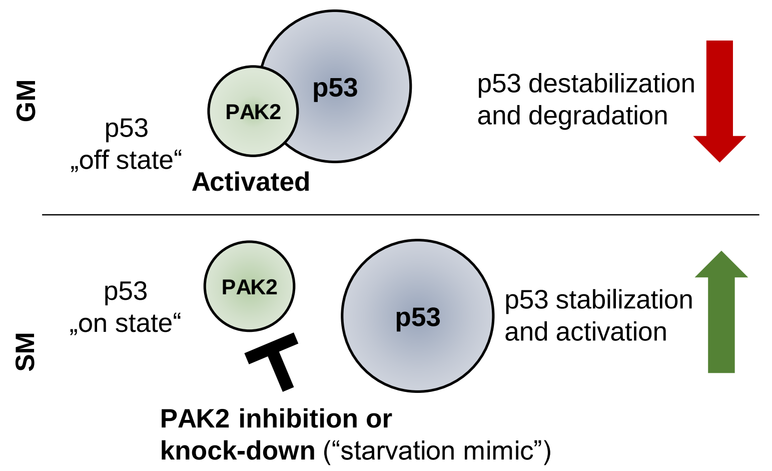

Supplemental Figure S3.

(A) Venn diagram showing overlaps between previously found PAK2 interactors derived from the BioGRID database and proteins interacting with p53 under GM conditions found with proximity-labelling mass spectrometry (BioID).
(B) Predicted 14-3-3 binding sites in PAK2 and p53 (Madeira et al. 2015).
(C) Intensity ratio of p53 DBD residues in bound vs free state in NMR binding assay titration of PAK21-212 to p53DBD (pertaining to Fig 3C).
(D) NMR spectrum of 15N- labelled p53 transactivation domain (TAD, 1-94) with PAK2 N-terminal domain (1-212) showing no chemical shift perturbation.
(E) Proposed model of p53 activation mediated by dissociation from PAK2.
.

A

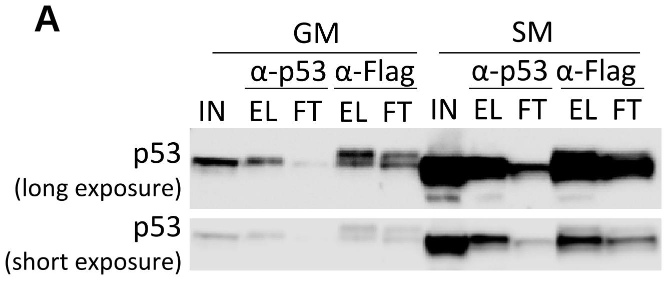

B

iBAQ intensities p53-DO1 pulldown

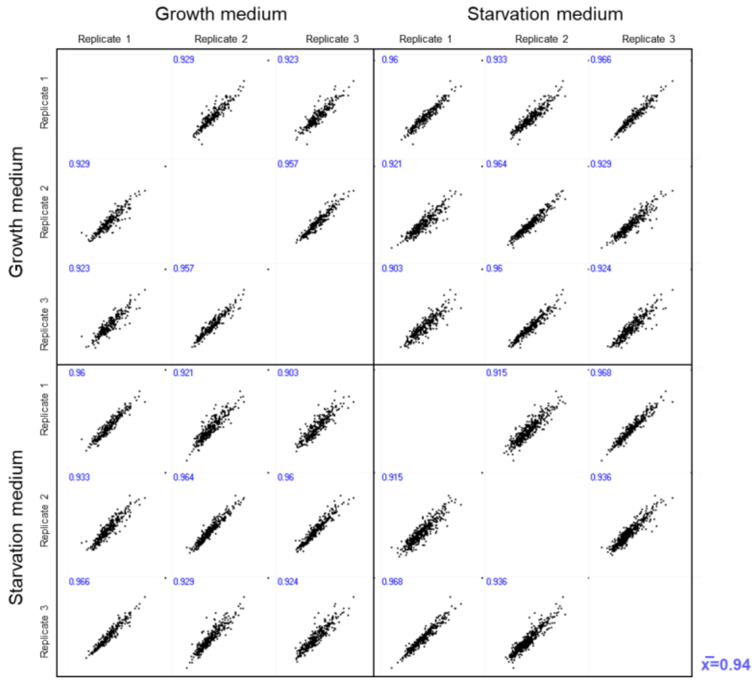

C

RIME PCA analysis

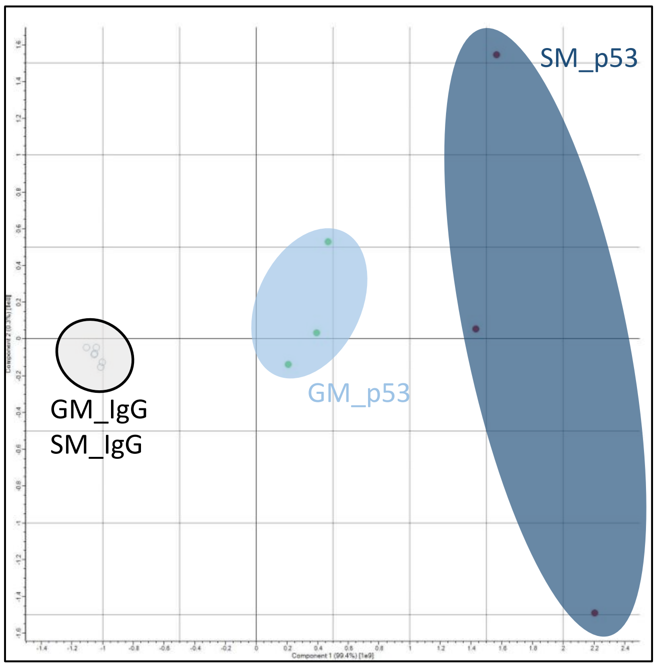

D **p53 peptide count per sample**

Length	Sequence	Start position	End position	GM-DO1-1	GM-DO1-2	GM-DO1-3	GM-IgG-1	GM-IgG-2	GM-IgG-3	SM-DO1-1	SM-DO1-2	SM-DO1-3	SM-IgG-1	SM-IgG-2	SM-IgG-3
13	ALPNNTSSSPQPK	307	319			1				1	1	1			
9	ELNEALELK	343	351	1	1	1				1	1	1			
13	KPLDGEYFTLQIR	321	333		1					1	1	1			
19	RPILTIITLEDSSGNLLGR	249	267			1				1	1				
7	TEEENLR	284	290	1	1	1						1			
9	TYQGSYGFR	102	110	1	1	1				1	1	1			

Supplemental Figure S4.

(**A**) Western blot showing pulldown experiments validating p53 DO1 antibody for endogenous p53 pulldown in comparison to FLAG-p53 pulldown with FLAG beads. Lower band: p53; upper band: co-eluted IgG heavy chain. IN…input, EL...eluate, FT…flow through.
(**B**) Multi-scatter plot to verify replicate quality. Average Pearson correlation=0.94.
(**C**) PCA (principal component analysis) of RIME samples.
(**D**) Number of p53 peptides found per replicate with RIME analysis.

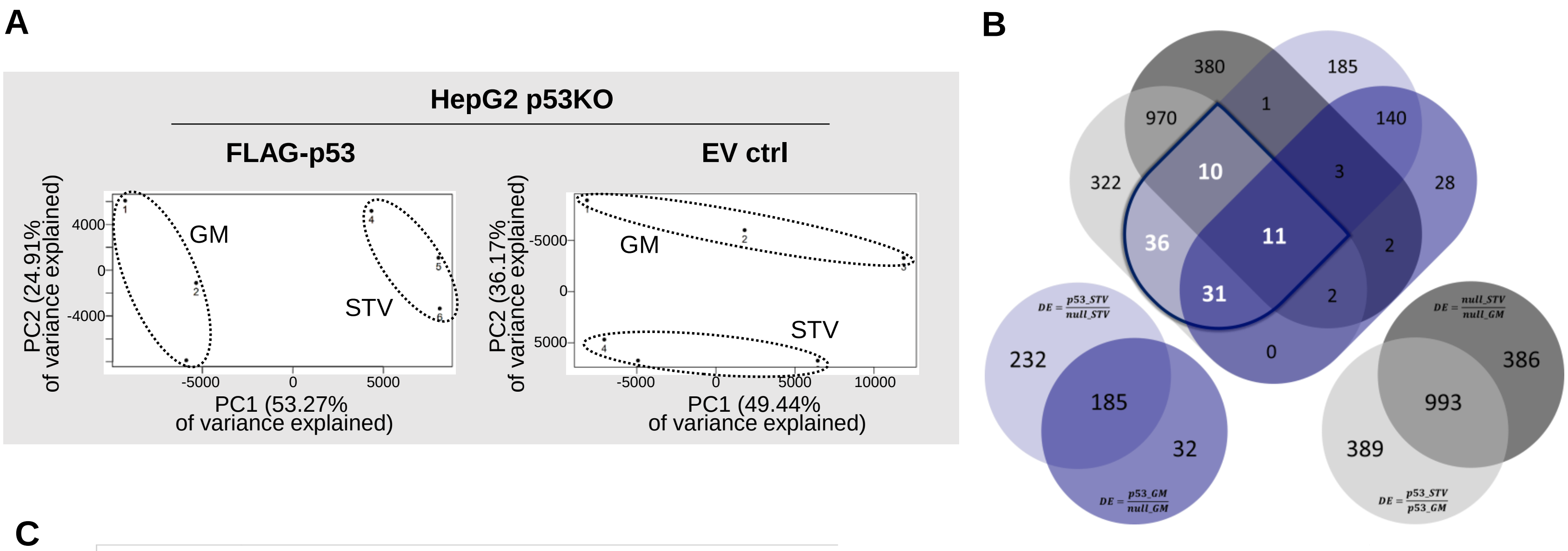

C

REACTOME pathway - known p53 targets

Gene Set	Description	Size	Expect	Ratio	P Value	FDR
**R-HSA-6791312**	**TP53 Regulates Transcription of Cell Cycle Genes**	**49**	**0.10678**	**46.823**	**5.53E-08**	**0.000086479**
**R-HSA-6804115**	**TP53 regulates transcription of additional cell cycle genes...**	**21**	**0.045765**	**87.404**	**1.00E-07**	**0.000086479**
**R-HSA-3700989**	**Transcriptional Regulation by TP53**	**365**	**0.79543**	**10.057**	**5.90E-07**	**0.00033956**
R-HSA-6803211	TP53 Regulates Transcription of Death Receptors and Ligands	12	0.026151	76.478	0.0002959	0.071388
R-HSA-5633007	Regulation of TP53 Activity	160	0.34868	11.472	0.00035977	0.071388
R-HSA-69563	p53-Dependent G1 DNA Damage Response	66	0.14383	20.858	0.00037829	0.071388
R-HSA-69580	p53-Dependent G1/S DNA damage checkpoint	66	0.14383	20.858	0.00037829	0.071388
R-HSA-6804759	Regulation of TP53 Activity through Association with Co-factors	14	0.03051	65.553	0.0004069	0.071388
R-HSA-6804116	TP53 Regulates Transcription of Genes Involved in G1 Cell Cycle Arrest	14	0.03051	65.553	0.0004069	0.071388
R-HSA-69615	G1/S DNA Damage Checkpoints	68	0.14819	20.244	0.00041313	0.071388

REACTOME pathway - putative novel p53 targets

Gene Set	Description	Size	Expect	Ratio	P Value	FDR
R-HSA-447038	NrCAM interactions	7	0.015918	125.64	0.00010336	0.1786
R-HSA-1266738	Developmental Biology	1074	2.4423	2.8662	0.0081193	1
R-HSA-193670	p75NTR negatively regulates cell cycle via SC1	6	0.013644	73.292	0.01357	1
R-HSA-190370	FGFR1b ligand binding and activation	6	0.013644	73.292	0.01357	1
R-HSA-205025	NADE modulates death signalling	6	0.013644	73.292	0.01357	1
R-HSA-112315	Transmission across Chemical Synapses	227	0.5162	5.8117	0.014241	1
R-HSA-442720	CREB phosphorylation through the activation of Adenylate Cyclase	7	0.015918	62.821	0.015814	1
R-HSA-447043	Neurofascin interactions	7	0.015918	62.821	0.015814	1
R-HSA-190371	FGFR3b ligand binding and activation	7	0.015918	62.821	0.015814	1
R-HSA-418886	Netrin mediated repulsion signals	8	0.018192	54.969	0.018054	1

Gene ontology Biological process - known p53 targets

Gene Set	Description	Size	Expect	Ratio	P Value	FDR
**GO:0072331**	**signal transduction by p53 class mediator**	**218**	**0.50051**	**15.984**	**2.53E-08**	**0.000021535**
**GO:0042770**	**signal transduction in response to DNA damage**	**131**	**0.30076**	**19.949**	**4.69E-07**	**0.00019922**
**GO:0007050**	**cell cycle arrest**	**240**	**0.55102**	**12.704**	**9.97E-07**	**0.00028238**
**GO:0000075**	**cell cycle checkpoint**	**216**	**0.49591**	**12.099**	**8.5886E-06**	**0.0018251**
**GO:0044843**	**cell cycle G1/S phase transition**	**247**	**0.56709**	**10.58**	**0.000018419**	**0.0031312**
**GO:0045930**	**negative regulation of mitotic cell cycle**	**256**	**0.58775**	**10.208**	**0.000022547**	**0.0031941**
**GO:0010948**	**negative regulation of cell cycle process**	**278**	**0.63826**	**9.4006**	**0.000035839**	**0.0043519**
**GO:0097193**	**intrinsic apoptotic signaling pathway**	**285**	**0.65433**	**9.1697**	**0.000041189**	**0.0043763**
GO:0045862	positive regulation of proteolysis	343	0.78749	7.6191	0.00011487	0.010849
GO:0045787	positive regulation of cell cycle	374	0.85867	6.9876	0.00018419	0.015481

Gene ontology Biological process - putative novel p53 targets

Gene Set	Description	Size	Expect	Ratio	P Value	FDR
GO:0035455	response to interferon-alpha	20	0.044567	44.876	0.00089225	0.53348
GO:0060560	developmental growth involved in morphogenesis	225	0.50138	7.9779	0.0015023	0.53348
GO:0006929	substrate-dependent cell migration	29	0.064623	30.949	0.0018829	0.53348
GO:0048871	multicellular organismal homeostasis	470	1.0473	4.774	0.0035893	0.54628
GO:0061564	axon development	490	1.0919	4.5792	0.0042868	0.54628
GO:2000147	positive regulation of cell motility	493	1.0986	4.5513	0.0043993	0.54628
GO:0048483	autonomic nervous system development	46	0.10251	19.511	0.0046877	0.54628
GO:0051302	regulation of cell division	158	0.35208	8.5207	0.0051415	0.54628
GO:0021675	nerve development	77	0.17158	11.656	0.012695	1
GO:0007265	Ras protein signal transduction	437	0.9738	4.1076	0.015558	1

Supplemental Figure S5.

(A) PCA (principal component analysis) of RNAseq samples from HepG2 p53KO cells either expressing FLAG-p53 or EV ctrl.
(B) Venn diagram showing overlaps of DEanalysis (DEseq2, cutoff p-value<0.05, Benjamini-Hochberg corrected) between each sample group. White numbers indicate groups of transcripts responsive to p53 re-expression and starvation stimulus. Group comparisons indicated within the colored circles.
(C) Mapping of known p53 targets to gene ontology biological processes or to REACTOME pathways (WebGestalt).
(D) Chromatin immunoprecipitation followed by quantitative PCR (ChIP-qPCR) was performed from crosslinked HepG2 cells kept in starvation or growth medium (SM vs GM). Anti-p53 antibody (DO1 clone) and anti-IgG antibodies (unspecific pulldown control) were used for pulldown from sheared chromatin. Fold-enrichment between SM and GM was calculated through relating values from pulldown samples to input and IgG values. qPCR primers were designed in predicted p53 binding sites near the transcription start sites. CDKN1A and BTG2 served as positive control and ARG2 (a gene not differentially regulated through p53 overexpression in our RNA-seq data set) served as negative control.
(E) HepG2 cells treated with the MDM2 inhibitor nutlin-3a for 24 hours. PHLDA3 is a known nutlin-inducible p53 target gene. Data is shown as mean ± SEM. N=3, multiple t-test (unpaired) was performed.

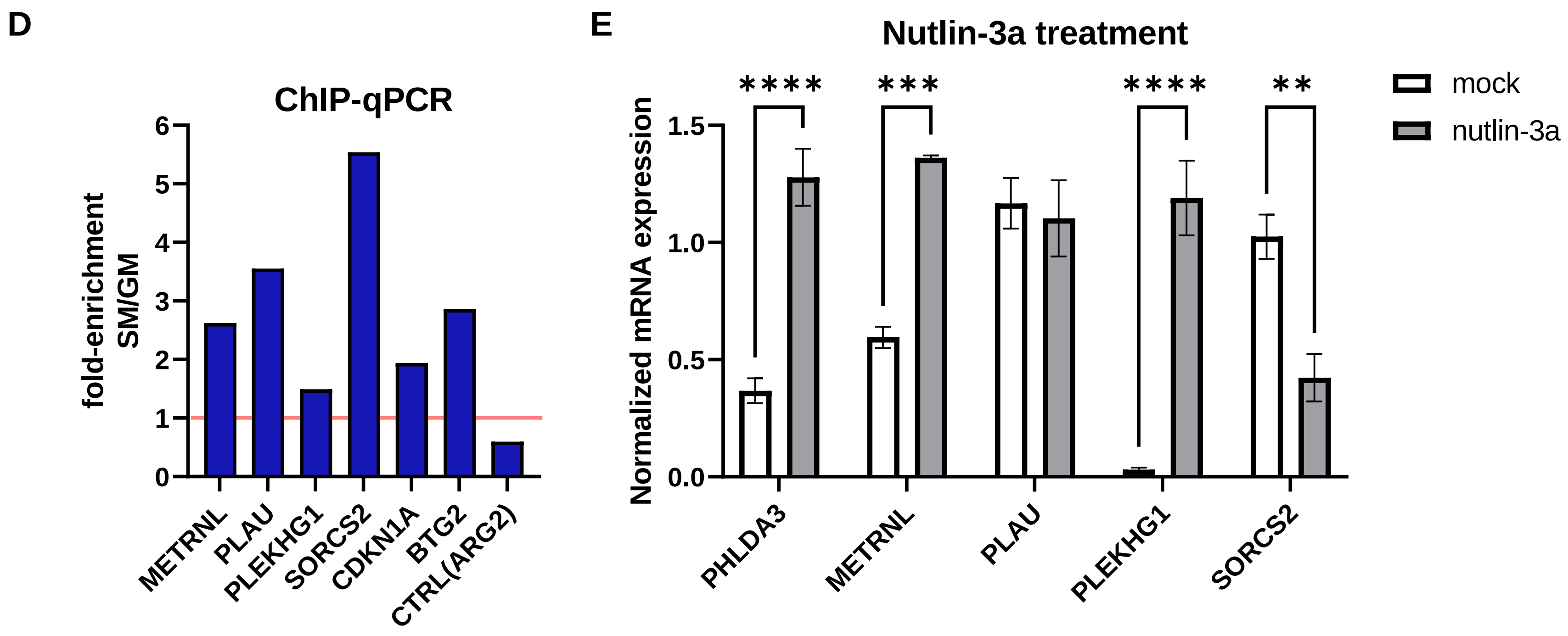

Supplemental Figure S5 (cont).

(legend on previous page)
